# Transcriptome and Complexity-Reduced, DNA-Based Identification of Intraspecies Single-Nucleotide Polymorphisms in the Polyploid *Gossypium hirsutum* L.

**DOI:** 10.1534/g3.114.012542

**Published:** 2014-08-07

**Authors:** Qian-Hao Zhu, Andrew Spriggs, Jennifer M. Taylor, Danny Llewellyn, Iain Wilson

**Affiliations:** CSIRO Plant Industry, Canberra, ACT 2601, Australia

**Keywords:** cotton genomics, QTL mapping, RNA-sequencing, SNP detection, SNP genotyping

## Abstract

Varietal single nucleotide polymorphisms (SNPs) are the differences within one of the two subgenomes between different tetraploid cotton varieties and have not been practically used in cotton genetics and breeding because they are difficult to identify due to low genetic diversity and very high sequence identity between homeologous genes in cotton. We have used transcriptome and restriction site−associated DNA sequencing to identify varietal SNPs among 18 *G. hirsutum* varieties based on the rationale that varietal SNPs can be more confidently called when flanked by subgenome-specific SNPs. Using transcriptome data, we successfully identified 37,413 varietal SNPs and, of these, 22,121 did not have an additional varietal SNP within their 20-bp flanking regions so can be used in most SNP genotyping assays. From restriction site−associated DNA sequencing data, we identified an additional 3090 varietal SNPs between two of the varieties. Of the 1583 successful SNP assays achieved using different genotyping platforms, 1363 were verified. Many of the SNPs behaved as dominant markers because of coamplification from homeologous loci, but the number of SNPs acting as codominant markers increased when one or more subgenome-specific SNP(s) were incorporated in their assay primers, giving them greater utility for breeding applications. A *G. hirsutum* genetic map with 1244 SNP markers was constructed covering 5557.42 centiMorgan and used to map qualitative and quantitative traits. This collection of *G. hirsutum* varietal SNPs complements existing intra-specific SNPs and provides the cotton community with a valuable marker resource applicable to genetic analyses and breeding programs.

Molecular markers genetically linked to agronomically important traits or genes of interest are valuable tools for increasing the efficiency of crop genetic improvement through marker-assisted selection. A number of different types of molecular markers, including restriction fragment length polymorphism, random amplified polymorphic DNA, simple sequence repeat (SSR), diversity arrays technology, amplified fragment length polymorphism, and single-nucleotide polymorphism (SNP), have been developed and used successfully in genetic studies of both simple and complex quantitative traits. SNPs are the most abundant type of molecular markers in plants, even in species that are restricted in their genetic diversity such as many of our crops (Ganal *et al.* 2009). Before the widespread application of efficient next-generation sequencing (NGS) technologies, large-scale SNP discovery was time-consuming, expensive, and impractical in species without a reference genome. Within the last decade, several different NGS approaches have been applied in large-scale SNP discovery efforts in both model plant species and agriculturally important crop species. These approaches include whole-genome DNA resequencing, transcriptome or RNA sequencing (RNA-seq), complexity-reduced-DNA sequencing (Davey *et al.* 2011), and targeted sequence capture and resequencing (Mascher *et al.* 2013). Among these approaches, RNA-seq, restriction site−associated DNA (RAD) sequencing (Baird *et al.* 2008), and genotyping-by-sequencing (GBS; Elshire *et al.* 2011; Poland *et al.* 2012) are particularly useful in plant species lacking a reference genome (Wang *et al.* 2012a; Yang *et al.* 2012). The advances in SNP discovery using NGS together with the development of high-throughput SNP genotyping technologies have made SNPs the marker of choice in a wide range of plant studies, although their use has lagged behind in polyploid species compared with diploids. These applications of SNPs include, but are not limited to, construction of high-density genetic linkage maps for dissecting quantitative trait loci (QTL; Bancroft *et al.* 2011; Wang *et al.* 2012a), investigation of genetic diversity and population structure of crop germplasm (Chen *et al.* 2011; Cavanagh *et al.* 2013), sequence assembly and genome comparison (Bancroft *et al.* 2011; Saintenac *et al.* 2013), determination of recombination breakpoints (Huang *et al.* 2009), and genome-wide association studies (Huang *et al.* 2012; Jiao *et al.* 2012; Riedelsheimer *et al.* 2012; Li *et al.* 2013).

In polyploid species, like wheat, oilseed rape, sugarcane, and cotton, SNP identification still remains a significant analytical challenge, although some progress is being made with new sequencing technologies and new bioinformatic algorithms (Kaur *et al.* 2012). Tetraploid cotton is the most important fiber crop in the world; however, large-scale identification and use of SNPs in cotton remains in its infancy because of several inherent species-specific limitations. First, *Gossypium hirsutum* (Upland cotton) and *G. barbadense* (Sea Island, Pima, or Egyptian cotton), the two cotton species with the largest areas of cultivation worldwide (90% and 6% of global production, respectively) are both allotetraploids. They originated from a relatively recent (1−2 million years ago) interspecific hybridization event between an A-genome−like ancestral African diploid species similar to modern *G. arboreum* or *G. herbaceum* and a D-genome−like Central American diploid species similar to modern *G. raimondii* (Wendel and Cronn 2003). The two subgenomes (A_t_ and D_t_, representing the A and D subgenome of tetraploid cotton, respectively) of tetraploid cotton have a very high (often >95%) sequence conservation between homeologous genes. This means that the genic contents of the two subgenomes often are difficult to distinguish from each other in short read sequences from tetraploid plants. Second, compared with varieties among other major crop species, Upland cotton varieties, including our Australian cotton varieties, have a relatively low DNA sequence diversity. The average frequency of SNPs in Upland cotton was reported to be from less than 0.01–0.04% (Rungis *et al.* 2005; Van Deynze *et al.* 2009). This is probably because the majority of Upland cotton now grown has gone through a number of severe genetic bottlenecks, initially during domestication and then through subsequent breeding, and are now largely generated from repeated use of just a few related genetic backgrounds (Rahman *et al.* 2002; Wendel *et al.* 2010). Finally, a reference A_t_D_t_ genome is not yet available, although the draft reference genomes of *G. raimondii* (D_5_) and *G. arboreum* (A_2_), the putative extant form of the contributor of the D_t_ and A_t_ genomes of the cultivated tetraploid cotton species, respectively, have recently been sequenced (Paterson *et al.* 2012; Wang *et al.* 2012b; Li *et al.* 2014).

Although there have been many reports on the identification of SNPs in cotton, the majority were either interspecific SNPs or, if intraspecific SNPs, were identified from the analysis of sequence data of a single or few genes (Small *et al.* 1999; An *et al.* 2007, 2008; Hsu *et al.* 2008). These SNPs have had little utility in breeding because they are generally not polymorphic among intraspecific breeding populations. Large-scale identification of SNPs in *G. hirsutum* has been relatively recent, and although the CottonGen Database (https://www.cottongen.org) lists some 56,961 *G. hirsutum* SNPs identified from Public expressed sequence tag (EST) data, no effort was put into identifying varietal variation and most are likely to be subgenome-specific SNPs. Few, if any, of those computationally predicted SNPs have been validated or mapped. The first dedicated effort in the public domain identified SNPs by sequencing a large number of amplicons from prescreened single copy loci using the traditional Sanger sequencing approach. More than a thousand SNPs were identified from a panel of diverse *Gossypium* germplasm, but only 245 SNPs from 124 loci were found among the 16 *G. hirsutum* accessions examined (Van Deynze *et al.* 2009).

Using a hypomethylated restriction-based genomic enrichment strategy and the 454 pyro-sequencing technology, Rai *et al.* (2013) recently identified 66,364 potential SNPs (again many likely to be subgenome-specific) among six Indian *G. hirsutum* lines, but only 30 of those were selected for validation using the Sequenom platform, so their utility remains to be further verified. Using a complexity-reduced, DNA sequencing approach, Byers *et al.* (2012) found 11,834 SNPs between a commercial *G. hirsutum* variety Acala Maxxa and a wild race cotton TX2094 (*G. hirsutum* race *yucatenense*) at the extreme of the diversity within *G. hirsutum*. Only a small proportion (<7%) of a subset consisting of 277 codominant markers assayed on a diverse panel of germplasm were found to be different between other domesticated varieties and Acala Maxxa, so not many of these SNPs are likely to be polymorphic between different breeding lines. A SNP genetic linkage map was constructed using 367 of the total of 1052 SNPs developed from this resource, validating the utility of the SNPs for introgressions from wilder sources of *G. hirsutum*, but not necessarily for normal intervarietal cotton breeding. Salmon *et al.* (2012) isolated and sequenced 500 pairs of homologous genes from Acala Maxxa and TX2094 using the recently developed targeted sequence capture approach; however, only 31 varietal SNPs were added to those previously identified between these two lines. These existing efforts have provided important early gains in cotton genomics and SNP discovery; however, there remains a strong need to develop robust SNP identification methodologies that will be effective for SNP discovery among elite varieties with relatively narrow or common pedigrees and to have more genuine varietal SNPs available for maker-assisted breeding in *G. hirsutum*.

In this study, we performed varietal SNP identification using transcriptomes of 18 *G. hirsutum* varieties and complexity-reduced, DNA sequences from two of those varieties using a novel approach and validated a significant subset (1363 SNPs) using the Sequenom or GoldenGate genotyping platform. This collection of *G. hirsutum*-specific varietal SNPs provides the cotton community with a valuable marker resource applicable to applied breeding targets and genetic analyses alike. Usage of these SNPs was demonstrated by construction of a *G. hirsutum* genetic linkage map containing 1244 SNP markers and the mapping of QTL for leaf shape, leaf trichome density, and pollen color.

## Materials and Methods

### Plant materials

In total, 18 different *G. hirsutum* varieties were used for SNP discovery in this study (Table 1). These varieties represent the core parental germplasm used in the Australian cotton breeding program. Apart from varieties developed in Australia, some were introduced from other countries, such as the United States (*e.g.*, Coker 315), India (*e.g.*, MCU-5), and China (*e.g.*, Lumein 14). All 18 varieties were used in transcriptome-based SNP identification, whereas MCU-5 [normal leaf shape, dense leaf trichome (603 ± 117/cm^2^), and yellow pollen color] and Siokra 1-4 [okra leaf shape, sparse leaf trichome (56 ± 9/cm^2^), and creamy pollen color] were also used in RAD-based SNP identification. Seeds of these varieties were provided by the cotton breeders of CSIRO Plant Industry, Narrabri, Australia. An F_7_ recombinant inbred line (RIL) population with 244 lines derived from MCU-5 × Siokra 1−4 (Lopez-Lavalle *et al.* 2012) was used in phenotyping for leaf shape, leaf trichome density and pollen color, and QTL mapping.

**Table 1:** Summary of RNA-sequencing results

ID	Variety	No. Raw Reads	No. Clean Reads Used in Alignment	No. Reads Uniquely Aligned to the D_5_ Genome	Percentage of Reads Uniquely Aligned to the D_5_ Genome, %
1	Sicot 70	54,444,448	49,252,968	20,404,115	41.4
2	Delta Opal	54,355,560	45,831,902	19,641,863	42.9
3	Siokra 1-4	50,682,422	47,158,057	19,764,911	41.9
4	Coker 315	53,221,990	48,937,618	21,342,354	43.6
5	Namcala	52,263,376	47,729,846	19,799,540	41.5
6	Sicala 40	52,380,470	48,147,300	20,275,813	42.1
7	Riverina Poplar	53,938,660	50,322,178	20,439,654	40.6
8	Sicot 189	53,570,564	49,909,281	18,275,447	36.6
9	Tamcot SP37	50,145,774	46,651,697	17,888,250	38.3
10	Sicot 81	54,109,928	49,862,743	19,918,393	39.9
11	Sicala V2	54,120,492	49,735,996	20,093,922	40.4
12	Sicot F-1*^a^*	27,743,884	27,155,816	13,531,837	49.8
13	MCU-5	57,351,294	52,660,801	21,957,187	41.7
14	Sicot 71	56,740,222	51,769,995	21,794,855	42.1
15	DP 16	56,189,338	51,932,005	22,177,646	42.7
16	DP 90	57,735,296	52,237,532	20,658,109	39.5
17	Sicala 3-2	54,833,166	50,027,369	20,646,722	41.3
18	Lumein 14	55,679,204	49,876,877	19,429,651	39.0

a Single-end reads only.

### Callus preparation, RNA extraction, and transcriptome sequencing

Callus induction was performed essentially using the procedures previously described (Cousins *et al.* 1991) except that the explants used were cotyledons. Total RNA was isolated from callus using the hot borate method (Wan and Wilkins 1994), tested for quality (with an RNA integrity number or RIN score >7) using the Bioanalyzer 2100 (Agilent Technologies), and submitted to Beijing Genomics Institute (BGI Hong Kong) for transcriptome sequencing according to their in-house protocols (RNA normalized using the Duplex-Specific thermostable nuclease enzyme). Sequencing was done using a HiSeq2000 instrument (Illumina) to generate 90-bp paired-end short reads.

### Generation of RAD sequencing libraries

The RAD sequencing libraries were generated according to the procedures reported by Baird *et al.* (2008) using *Eco*RI−, *Ape*kI−, or *Sbf*I−digested cotton genomic DNA, which was isolated from young leaves of MCU-5 and Siokra 1−4 using the DNeasy Plant Mini Kit (QIAGEN) according to the manufacturer’s instructions. Approximately 500 ng of DNA was used in digestion and ligation. To sequence the six libraries together in a single lane, Adaptor 1 with two different index sequences (Supporting Information, Table S1) was used for MCU-5 and Siokra 1−4. Single end reads with a length of 100 bp were generated using a HiSeq2000 (Illumina) at the Australian National University (Canberra, Australia).

### SNP discovery

After adaptor trimming and removal of low-quality reads, RNA-seq reads were stringently aligned against the cotton D_5_ genome (*G. raimondii*; ftp://ftp.jgi-psf.org/pub/compgen/phytozome/v9.0/Graimondii/) using Biokanga (http://www.biokanga.sourceforge.net) with the following settings: ≤4 bp of substitutions, ≤5 bp of microInDels, splice-junction detection for introns up to 1 kb in size, with 5′ and 3′ ends trimmed until edge bases matched the reference, polymerase chain reaction (PCR) differential amplification artifact reduction applied, and no indeterminate bases allowed. Only the reads with a single best unique alignment were used in the following SNP identification processes. The alignment results were written in SAM format.

A custom C++ application, developed in-house, was used to predict bialleic SNPs based on these alignments. The program used a sliding window approach to identify regions with enough read density for SNP calling. The observed SNPs within each variety and all possible permutations of their combinations in these regions were processed in an iterative strategy to identify well-supported, subgenome SNPs. These regions with discriminated genome SNPs were then used in varietal comparisons to identify varietal SNPs. The detailed bioinformatic algorithm will be described in another publication dealing with both the methodology and implementation (A. Spriggs, S. Stephen, Q.-H. Zhu, D. Llewellyn, I. Wilson, J. M. Taylor, unpublished data).

For the RAD sequencing data, after removing adaptor-ligated and low quality reads, we assigned the remaining reads to MCU-5 or Siokra 1−4 based on the index sequences, and the sorted reads were then further separated based on the restriction sites of *Eco*RI, *Ape*KI, and *Sbf*I. After further removing the index sequence, all reads were 3′ trimmed to a length of 78 bp. For each variety, identical short reads were collapsed into a sequence tag, and then the unique sequence tags with a read depth ≥4 (we found that sequencing errors could be effectively removed while keeping a maximum number of informative sequence tags when using this read depth threshold) from each variety were together aligned to the *G. raimondii* genome using the CLC Genomics Workbench (version 6.0.4; http://www.clcbio.com/products/clc-genomics-workbench/) with the following parameter settings: mismatch cost, 2; insertion and deletion cost, 3; length fraction, 0.5; similarity fraction, 0.95; and nonspecifically matched reads ignored.

We found that in the majority of regions with sequence tags mapped, only four tags were aligned, two from each variety, potentially one from the A_t_ genome, and another from the D_t_ genome. This alignment result was then used to call the potential SNPs using the “quality-based variation detection” model implemented in the CLC Genomics Workbench with the following settings: default read quality filters (*i.e.*, neighborhood radius, 5; maximum mismatch count, 2; minimum neighborhood quality, 15; minimum central quality, 20); minimum tag coverage, 4; minimum variant frequency, 25%; maximum expected alleles, 2. The results were then filtered using coverage (4) and allele frequency (25% and 75%) to get the first set of potential varietal SNPs. The rationale for using these filters are that the region with a potential varietal SNP should be covered by two sequence tags from each variety and that the potential varietal SNP should have a 1:3 ratio. The alignment status of the filtered SNPs were manually checked to further remove false-positive results and to make sure that a putative varietal SNP is always flanked by at least one genome-specific SNP because this information could not be obtained by filtering. All SNPs reported in this paper are shown in File S1 and File S2, and they have also been submitted to CottonGen (http://www.cottongen.org/).

### SNP validation

Selected putative varietal SNPs initially were analyzed by converting them to cleaved amplified polymorphic sequence (CAPS) markers. Primers were designed based on the aligned reads or the genome sequence of *G. raimondii* to amplify a ~200-bp PCR product using the varieties from which the SNP was identified. For each pair of primers, PCR conditions were optimized to amplify a single band. The PCR products were then digested with an appropriate restriction enzyme for 2−3 hr at the appropriate temperatures and fractionated on a 2% agarose gel to visualize the digestion products.

Sequenom SNP assays were performed using the standard procedure at the Australian Genome Research Facility (Melbourne, Australia). Primers were designed based on 100-bp flanking sequences of the putative varietal SNP and aligned against the *G. raimondii* genome sequence by Blastn to confirm their uniqueness. Illumina GoldenGate SNP assays were performed by Beijing Genomics Institute (BGI Hong Kong). Then, 100-bp flanking sequences of 1652 putative varietal SNPs (from both transcriptome and complexity-reduced DNA) identified between MCU-5 and Siokra 1−4 and evenly distributed on 13 *G. raimondii* scaffolds were submitted to Illumina for marker design suitability ranking, before the selection of SNPs to assay. Because the RAD reads were only 78-bp long, *G. raimondii* genomic sequence was used to extend the flanking sequences (up to 100 bp on each side of the varietal SNP to conform to their required design pipeline). Of these SNPs, 1632 SNPs were selected for Oligo Pool Assay synthesis and used to genotype MCU-5, Siokra 1−4, and 244 F_7_ RILs derived from MCU-5 x Siokra 1-4. Of these, 1572 and 60 had a suitability ranking score >0.6 and 0.4−0.6, respectively, and 1521 had a SNP call in ≥95% of the samples analyzed and were kept for further analysis.

### Phenotyping

The leaf shape of each individual plant of the F_7_ RIL population was classified as okra, intermediate, or normal. Pollen color was classified as yellow or creamy. For leaf trichome density, three 57-mm^2^ leaf discs were collected from the flanking area of the main vein of the eighth leaf, treated, and observed (the adaxial surface or the upper side of a leaf) as previously described (Pomeranz *et al.* 2013) using a Leica MZFL III dissector with an additional polarized light filter. Each branch of a multibranched trichome was counted as a separate trichome. Average number of the three observations from each plant was converted to number of trichome/cm^2^ and used in QTL mapping.

### Linkage group construction and QTL analysis

The software ICIM (*i.e.*, inclusive composite interval mapping) (Li *et al.* 2007) was used to construct the genetic linkage map and to perform QTL mapping. The Kosambi mapping function was selected to convert a recombination frequency to genetic distance (cM). Linkage groups and marker orders were determined by using a logarithm of the odds score of 15. Only linkage groups with at least four SNP markers were kept and used in QTL mapping. QTL mapping was performed by using the ICIM-Add method of the program (ICIM) and only significant QTL above the permutation (1000 times) threshold were reported. Graphical representations were generated using MapChart (Voorrips 2002). Assignment of linkage group to A_t_ or D_t_ subgenome was based on comparison of sequences containing RNA-seq−derived SNPs with those of the A_2_ genome (*G. arboretum*, our own unpublished callus transcriptome data; data not shown) and the D_5_ reference genome (*G. raimondii*; Paterson *et al.* 2012).

## Results

### Transcriptome-based SNP discovery in tetraploid cotton

To identify varietal SNPs in *G. hirsutum*, we sequenced individual transcriptomes of 18 *G. hirsutum* varieties using messenger RNA isolated from undifferentiated callus derived from cotyledons. This tissue was chosen because a substantial proportion of the genome is transcribed in callus while avoiding the highly expressed genes involved in photosynthesis that would otherwise dominate sequence reads from most vegetative tissues. After trimming and filtering, 45.8−52.7 M high-quality sequence reads (90-bp paired-end) from each of the individual varieties were retained for alignment, except for Sicot F-1, for which 27.2 M processed single-end reads were used in alignment (Table 1). Reads were aligned to the *G. raimondii* reference D_5_-genome sequence using a K-mer Adaptive Next Generation Aligner, Biokanga (http://www.biokanga.sourceforge.net), with the parameters detailed in the section *Materials and Methods*. Approximately 13.5−22.2 M (36.6–49.8%) reads originating from transcripts of both A_t_ and D_t_ genomes were uniquely aligned and used in SNP identification.

As an allotetraploid, cotton has two types of SNPs: i) the more abundant subgenome-specific SNPs, which are polymorphisms between homeologous loci from the A_t_ and D_t_ subgenomes but mostly nonpolymorphic between varieties; and ii) varietal or allelic SNPs (also called hemi-SNPs), which are polymorphisms in only the A_t_ or D_t_ genome between two different varieties. Accurate identification of varietal SNPs in cotton using RNA-seq data, however, is complicated by: i) the lack of availability of transcriptome or genome reference sequences for both the subgenomes; ii) the presence of genome-specific SNPs at frequencies much higher than varietal SNPs; and iii) the possibility of unequal or differential allelic expression of homeologs from the A_t_ and D_t_ subgenomes, making it difficult to be certain that all alleles present have been observed for the purposes of robustly assigning a sequence difference to a varietal type.

**Figure 1:**
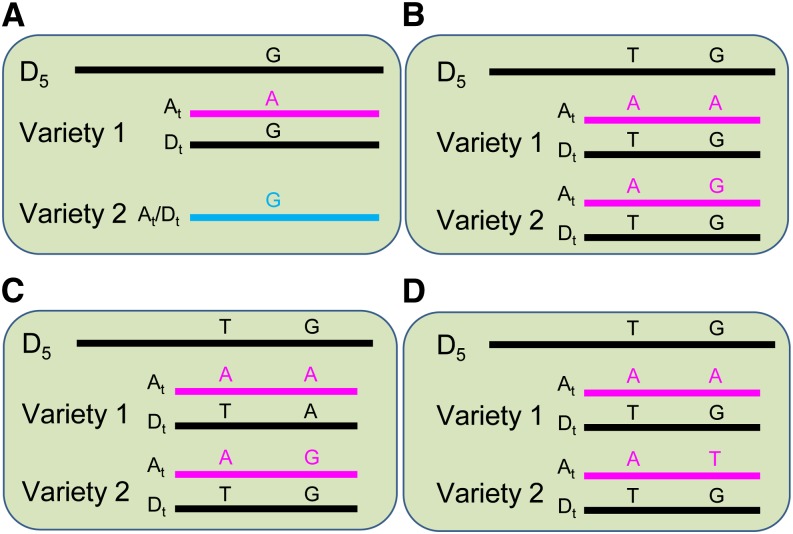
Varietal single-nucleotide polymorphisms (SNPs) can be more confidently called when it is flanked by a subgenome-specific SNP. (A) The sequence alignment pattern of two varieties when there is a putative varietal SNP (A/G) located on the A_t_ genome but with no subgenome-specific SNP nearby to help assign reads to their correct subgenome. In this case, the consensus sequence of variety 2 could be contributed by reads from both the A_t_ and D_t_ subgenomes with a G at the SNP position or just from one subgenome as a result of differential expression of homeologs or even by chance due to low sequence depth. It is therefore difficult to determine with any confidence whether or not A/G is a true varietal SNP. (B) The sequence alignment pattern of two varieties when there is a subgenome-specific SNP (A/T) flanking the putative varietal SNP (A/G). In this case, A/G is a quite confidently called varietal SNP due to presence of both the A_t_ and D_t_ alleles in sequence reads from both varieties. (C) A/G is a simple SNP and acts as a codominant marker in both homeologs. (D) A/T of the A_t_ genome is a candidate codominant SNP marker because of the presence of the flanking subgenome-specific SNPs. D_5_ represents *G. raimondii* genome sequence. Bars represent genomic DNA or consensus cDNA sequences derived from RNA-seq reads.

In a short sequence read alignment pattern looking like that shown in Figure 1A, for example, there could be an A/G varietal SNP on the A_t_ subgenome between variety 1 and 2, but only if there is definitely a G in this position in both the A_t_ and D_t_ alleles of variety 2. Alternatively, the nucleotide G detected in variety 2 may simply reflect the sequencing depth being by chance insufficient to detect the A_t_ allele or the D_t_ allele because of their differential expression and/or technical issues related to library preparation and sequencing. These two scenarios cannot be distinguished with any great confidence, unless the sequencing depth is very large, and would otherwise result in a very high false-positive rate for varietal SNP calls. If there is a genome-specific SNP (*e.g.*, A/T in Figure 1B), however, observed in the flanking region of the putative varietal SNP (A/G in Figure 1B), it can be used as a guide to resolve the reads from coexpressed homeologs from the A_t_ and D_t_ subgenomes and more confidently call the adjacent A/G as a varietal SNP present in the A_t_ genome.

**Figure 2:**
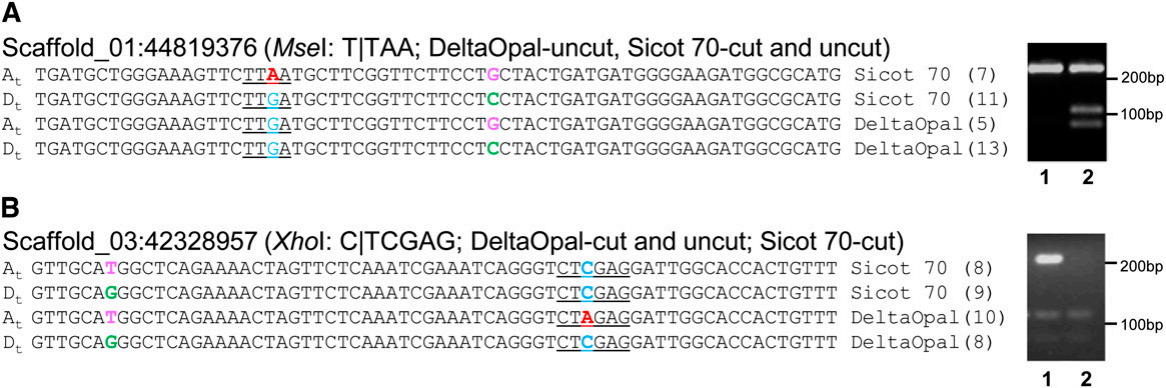
Verification of transcriptome-based predicted varietal SNPs by the CAPS method. The subgenome-specific SNPs are shown in pink and green, and the varietal SNPs are shown in red and blue. The restriction sites used for cleavage of the generated polymerase chain reaction fragments are underlined. The numbers in parentheses after the variety names represent the number of RNA-seq reads with identical sequences to that shown. Lanes 1 and 2 of the agarose gel represent DeltaOpal and Sicot 70, respectively. DNA size markers are indicated in bp. Sub-genome designations (A_t_ and D_t_) are inferred by comparison to *G. raimondii* and *G. arboreum* sequences.

Using this rationale, we deployed an analytical approach to identify only the varietal SNPs among our RNA-seq data that were flanked by at least one subgenome-specific SNP. The approach was first tested using the short reads from four varieties (MCU-5, Siokra 1−4, DeltaOpal and Sicot 70), and this identified 4894 varietal SNPs. Ten of the predicted varietal SNPs and 10 equivocal SNPs without a supporting adjacent subgenome-specific SNP were selected for validation by converting them to CAPS markers. Of the 10 predicted varietal SNPs with flanking subgenome-specific SNP(s), 7 were confirmed to be polymorphic. Two such examples are shown in Figure 2. In contrast, of the 10 equivocal SNPs, a polymorphism was confirmed in only one case, supporting our hypothesis. We then extended the analysis to call varietal SNPs by parallel processing the RNA-seq data from all 18 varieties. In total, 37,413 nonredundant varietal SNPs were identified among these *G. hirsutum* varieties (File S1).

### Use of complexity reduced genomic DNA in SNP discovery

Transcriptome-based varietal SNPs are limited to expressed regions of the genome that are likely to be less polymorphic as they are often constrained by purifying selection. To identify SNPs located within nontranscribed and intronic regions and to test the feasibility of using DNA instead of RNA of tetraploid cotton in SNP identification, we created a RAD sequencing library using genomic DNA isolated from MCU-5 and Siokra 1−4 and digested with *Eco*RI, *Ape*KI, or *Sbf*I. In total, 95.5 M single-end reads (100 bp in length) were generated. After adaptor trimming and removal of low-quality reads, the remaining reads were separated based on the index sequences and restriction sites into seven groups (MCU-5-*Eco*RI, MCU-5-*Ape*KI, MCU-5-*Sbf*I, Siokra 1-4-*Eco*RI, Siokra 1-4-*Ape*KI, Siokra 1-4-*Sbf*I, and a set of others that lacked the index sequence and/or restriction site and so were discarded). Of the reads (76.7 M) with both index and restriction-site information, the majority (95.3%) were from *Eco*RI−digested DNA, with only 2.2 M (2.8%) and 1.4 M (1.9%) from *Ape*KI− and *Sbf*I−digested DNA, respectively. This was probably because *Ape*KI and *Sbf*I did not digest cotton DNA well in our hands and *Sbf*I is a rare cutter restriction enzyme (8-bp cutter). In the following analysis, we used only reads from *Eco*RI digested DNA.

**Figure 3:**
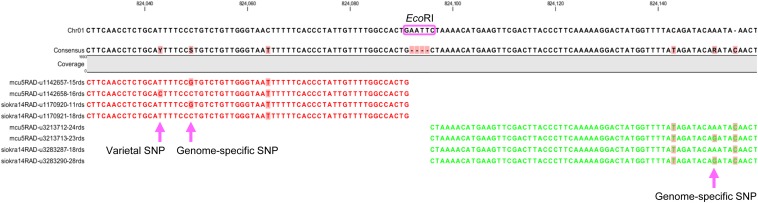
Use of the CLC Genomics Workbench in the identification of restriction-site associated DNA (RAD)-based single-nucleotide polymorphisms (SNPs). A screen shot showing RAD sequence tags aligned to the genome sequence of *G. raimondii*. The *Eco*RI restriction site used to generate the sequencing library is boxed. A varietal SNP (at position Chr01-824043) can be quite confidently called due to the presence of a subgenome-specific SNP (at position Chr01-824049) in the same reads.

We used the CLC Genomics Workbench to identify putative varietal SNPs in our RAD sequencing data. We investigated different combinations of the input sequence format and variation detection modules and found that potential varietal SNPs between two tetraploid cotton varieties could be quite accurately called using a nonredundant read set and the “quality-based variation detection” module by following the criteria detailed in the section *Materials and Methods*. An example is shown in Figure 3, where only two types of tags per genotype could be aligned to the *G. raimondii* genome on both sides of the *Eco*RI (GAATTC) restriction site. These tags from each genotype are distinguished from each other by the presence of subgenome-specific SNPs on the same sequence reads. The varietal SNP (C/T) shown has a 1:3 ratio among the four tags. Based on this type of alignment pattern and the filters described in Materials and Methods, we identified an additional 3,090 varietal SNPs between MCU-5 and Siokra 1-4 (File S2).

### Few varietal SNPs from transcriptome and RAD sequencing are overlapping

**Figure 4:**
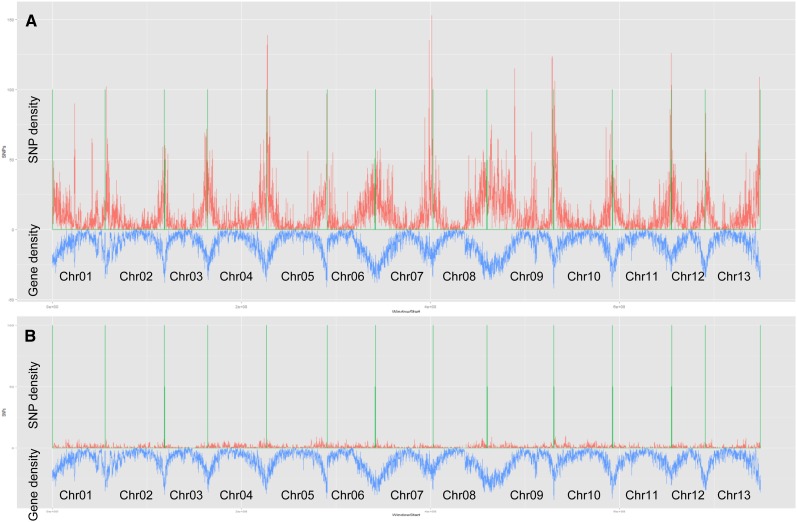
SNP and gene density across the 13 *G. raimondii* chromosomes. The orange peaks represent SNP density, which were generated by counting the number of SNPs in 50-kb sliding windows with a 25-kb overlapping region. Gene density in the same sliding windows was shown by blue peaks and mirrored on a single image. The green lines represent the positions of chromosome starts and/or ends as the graph shows all chromosomes joined together one after another. (A) RNA-seq−derived SNPs. (B) Restriction-site associated DNA−derived SNPs.

The varietal SNPs identified in this study were distributed across all *G. raimondii* chromosomes with an average density of 53 SNPs/Mb. Of the 13 chromosomes, Chr02 and Chr09 had the lowest (42 SNPs/Mb) and highest (78 SNPs/Mb) SNP density, respectively (Table 2). To examine the overlap in SNP calls between the transcriptome and RAD sequencing approaches, we used Blast (E value ≤1.0e-50) to align the flanking sequence (100 bp on each side) of all varietal SNPs identified by either approach between MCU-5 and Siokra 1−4 against the full set of predicted transcripts of *G. raimondii* (ftp://ftp.jgi-psf.org/pub/compgen/phytozome/v9.0/Graimondii/). Flanking sequences of the transcriptome- and RAD-derived varietal SNPs matched *G. raimondii* transcripts in 92.2% and 19.9% of cases, respectively. Only 43 (or 1.4%) of 3090 RAD-derived SNPs were overlapping with the transcriptome-derived SNPs. In addition, gene density was highly correlated with the density of transcriptome-derived SNPs but not with the density of RAD-derived SNPs that had a much more even distribution within each chromosome than transcriptome-derived SNPs (Figure 4). These results suggest that the RAD-derived varietal SNPs were mainly from nontranscribed regions and that these two approaches are complementary because they target different genomic features and that an even distribution of SNPs across the whole genome would be achieved by using both approaches.

**Table 2:** Distribution of SNPs across all chromosomes of *G. raimondii*

Chromosome	Total No. SNPs	Transcriptome Derived	RAD Derived	Chromosome Length, Mb	SNPs/Mb
1	2711	2467	244	55.9	48
2	2613	2385	228	62.7	42
3	1960	1860	100	45.8	43
4	3349	3003	346	62.2	54
5	3524	3188	336	64.1	55
6	2666	2426	240	51.1	52
7	4049	3863	186	61.0	66
8	3076	2883	193	57.1	54
9	5502	5223	279	70.7	78
10	3256	2870	386	62.2	52
11	2778	2601	177	62.7	44
12	1817	1681	136	35.4	51
13	3069	2844	225	58.3	53
Unassigned scaffolds	133	119	14	12.2	11
Total	40,503	37,413	3,090	761.4	53

SNP, single-nucleotide polymorphism; RAD, restriction site−associated DNA.

### Experimental validation of varietal SNPs

Some of the initial transcriptome based varietal SNPs were verified by conversion to CAPS markers as indicated previously. We extended the validation to two different high-throughput SNP genotyping platforms to verify a larger number of the predicted SNPs (Table 3). First, 65 transcriptome-derived varietal SNPs identified between Sicot 70 and DeltaOpal were analyzed using the Sequenom platform. Of the 62 that were successfully amplified, 45 (72.6%) were verified to be polymorphic between the two varieties. Second, 513 RAD-derived and 1119 transcriptome-derived varietal SNPs identified between MCU-5 and Siokra 1−4 were genotyped using the GoldenGate platform. A total of 467 RAD-derived and 1054 transcriptome-derived SNPs were successfully genotyped, and of these, 351 (75.2%) and 967 (91.7%), respectively, were confirmed to be polymorphic between the two varieties. These results suggest that a functional SNP assay can be designed for the majority of the varietal SNPs we identified. Although the SNPs used in validation were based on analysis of MCU-5 and Siokra 1−4 (*G. hirsutum*), 689 (52.3%) of the verified SNPs were also found to be polymorphic between two standard varieties (TM-1: *G. hirsutum*; 3−79: *G. barbadense*) commonly used in cotton genetics and generation of a number of mapping populations, suggesting that our SNPs should be useful more broadly in both cotton genetics and breeding.

**Table 3:** Validation of SNPs

Origin of SNPs	Genotyping Platform	No. SNPs Genotyped	No. Successful SNP Assay	No. SNPs Verified	Percentage of SNPs Verified
RNA-seq	Sequenom	65	62	45	72.6
RNA-seq	GoldenGate	1,119	1,054	967	91.7
RAD sequencing	GoldenGate	513	467	351	75.2

SNP, single-nucleotide polymorphism; RAD, restriction site−associated DNA.

### Use of subgenome-specific SNPs in assay primers to improve the frequency of SNPs behaving as codominant SNP assays

**Figure 5:**
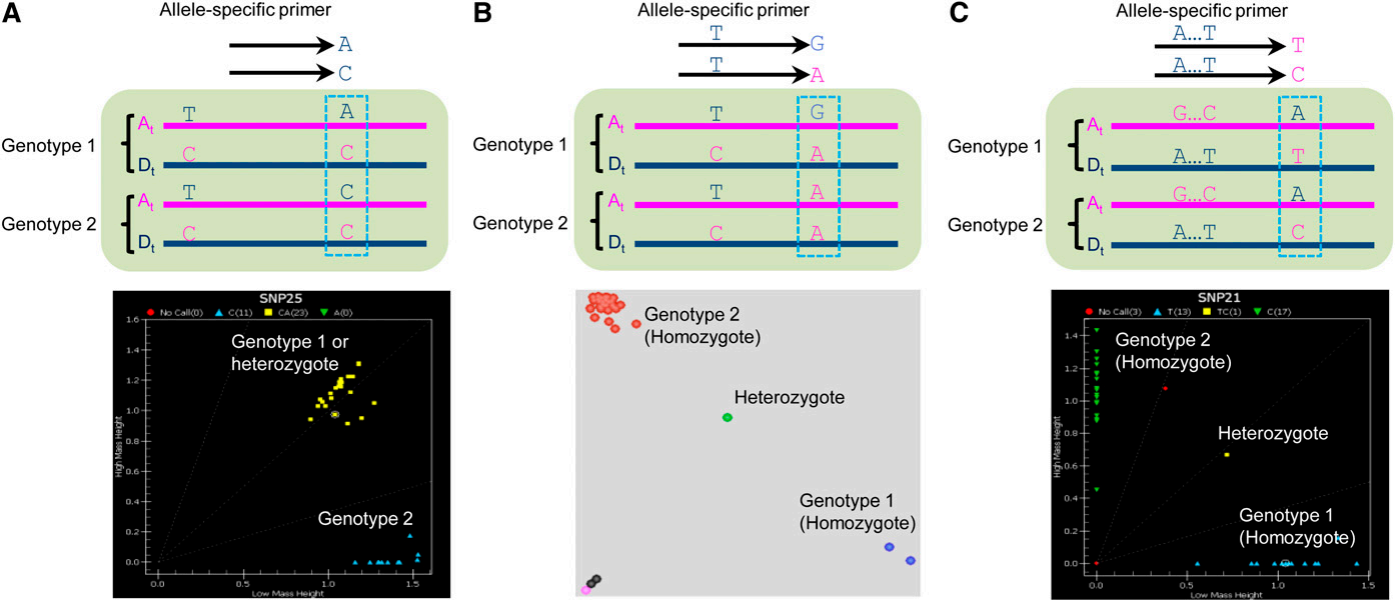
Depending on genotyping platforms and sequence context, varietal SNP markers can act as dominant or codominant SNP assays. (A) When subgenome-specific SNPs are not present in the allele-specific primers, both the A_t_ and the D_t_ subgenomes can be amplified and the varietal SNP assay is dominant, *i.e.*, it is unable to distinguish heterozygotes from homozygotes for one of the genotypes (Genotype 1 in this case for a Sequenom assay). (B) When a subgenome-specific SNP is present in the allele-specific primers, only the subgenome (the A_t_ subgenome in this case) with the subgenome-specific SNP is amplified; therefore, the varietal SNP assay is codominant, *i.e.*, heterozygotes can be separated from homozygotes in this kompetitive allele-specific PCR assay. (C) A codominant varietal SNP assay (T/C in this case) further enhanced by the presence of multiple subgenome-specific SNPs in the allele-specific primers in this Sequenom assay.

SNP genotype calling of most currently available SNP genotyping platforms, such as GoldenGate, kompetitive allele-specific PCR *(*i.e., KASP), and Sequenom, were developed primarily for diploid species. For polyploid species such as cotton, these platforms can be problematic when the SNP assay amplifies both homeologous (A_t_ and D_t_) copies of a locus, *i.e.*, they act as “dominant” SNP markers and are unable to discriminate between a heterozygous plant and one that is homozygous for one of the two possible alleles at that locus being assayed, such as Genotype 1 in Figure 5A. All genotyping platforms work well when the SNP assay amplifies only the homeologous copy carrying the SNP, *i.e.*, they act as codominant SNP assays as they would in a diploid species (Figure 5, B and C). Codominant SNP assays should be more useful in cotton breeding programs where it is essential to be able to identify individuals carrying all the homozygous alleles of interest in segregating populations.

There are theoretically two types of effective codominant SNP markers in a tetraploid using current genotyping platforms based on amplification of specific alleles with PCR: one as shown in Figure 1C, where the polymorphic SNP (A/G) between two varieties is a simple SNP identical in each subgenome, but different between varieties, and the other as shown in Figure 1D, where the polymorphic SNP (A/T) is only within one subgenome, in this case the A_t_ subgenome. In our pool of predicted varietal SNPs, only ~3% were of these types that should behave as codominant SNP assays, the rest potentially behaving as dominant SNP assays because the other subgenome homeologs carry the same nucleotide at the SNP position as one of the alleles (Figure 1B). If one or more subgenome-specific SNP(s) were present in the allele-specific and/or the universal primers used in a SNP assay, then a putative “dominant” varietal SNP marker with a sequence alignment pattern as shown in Figure 1B might behave like a true co-dominant SNP marker (Figure 5B) due to the destabilization of primer binding to one of the homeologs. As our varietal SNPs were identified based on the presence of flanking genome-specific SNP(s), some of them would have such genome-specific SNP(s) overlapping with the assay primers, so a portion of these SNPs would, in practice, function as co-dominant SNP markers. Consistent with this expectation, we found that 30.5% and 43.4% of transcriptome- and RAD-based SNPs, respectively, were effectively codominant SNP markers in the GoldenGate SNP genotyping assay.

To investigate the effectiveness of subgenome-specific SNP(s)-containing primers on the conversion of dominant SNP assays to co-dominant SNP assays, we analyzed the number and positions of subgenome-specific SNP(s) in both allele-specific and universal primers for the subset of 258 RAD-based SNPs that had their primers designed based on the 78-bp long RAD reads. The relationship between the number of subgenome-specific SNP(s) in the SNP assay primers and the percentage of assays behaving as co-dominant SNP markers are shown in Table 4. All 18 SNPs without subgenome-specific SNP in both the allele-specific and the universal primers behaved as dominant SNP markers, whereas all four SNPs with four subgenome-specific SNPs in either the allele-specific primer or the universal primer behaved as true codominant SNP markers. All four SNPs with three subgenome-specific SNPs in both the allele-specific and the universal primer also behaved as codominant SNP markers. Generally, although the numbers are small, the percentage of codominant SNP assays increased with an increasing number of subgenome-specific SNPs in the assay primers, irrespective of whether it was the allele-specific or the universal primer (Table 4). In addition, 30 codominant SNP assays and 77 dominant SNP assays had one genome-specific SNP in the allele-specific primer, the universal primer or both, and 50.0% (15/30) and 36.4% (28/77) of these SNPs had their subgenome-specific SNP within the 5-bp region of the 3′ end of the primers (Table 5). These results suggest that the number of genome-specific SNP(s) in the assay primers is positively correlated with the probability of a SNP assay performing as a codominant assay, and that a genome-specific SNP closer to the 3′ end of the assay primers is more useful for ensuring this behavior, but are not always effective.

**Table 4:** Effect of the number of subgenome-specific SNPs on the codominant behavior of SNP assay

	No. Subgenome-Specific SNPs in the Universal Primer					
	0	1	2	3	4	Overall for Allele-Specific Primer
No. subgenome-specific SNPs in the allele-specific primer						
0	0/18 (0.0)*^a^*	11/29 (37.9)	10/13 (76.9)	2/5 (40.0)	1/1 (100.0)	24/66 (36.4)
1	5/38 (13.2)	13/39 (33.3)	10/21 (47.6)	11/13 (84.6)	2/3 (66.7)	41/114 (36.0)
2	7/15 (46.7)	15/25 (60.0)	7/9 (77.8)	2/3 (66.7)		31/52 (59.6)
3	5/7 (71.4)	4/6 (66.7)	4/6 (66.7)	4/4 (100.0)		17/23 (73.9)
4	3/3 (100.0)					3/3 (100.0)
Overall for universal primer	20/81 (24.7)	43/99 (43.4)	31/49 (63.3)	19/25 (76.0)	3/4 (75.0)	

SNP, single-nucleotide polymorphism.

a Each cell has three numbers. The numbers before and after the forward slash represent the number of assays behaving as codominant and the total number of SNP assays in each group, respectively. The number in parentheses represents the percentage of codominant SNP assays.

**Table 5:** Distribution of single subgenome-specific SNP in assay primers of varietal SNPs behaving as codominant and dominant assays

Allele-Specific Primer	Codominant Assays					Dominant Assays				
	Universal Primer^a^					Universal Primer				
	I	II	III	IV	Null	I	II	III	IV	Null
I	2	1	1	0	3	1	1	2	5	9
II	1	1	0	2	0	1	2	2	2	4
III	1	0	0	1	1	1	2	0	1	8
IV	4	0	0	0	1	2	2	1	1	12
Null	2	3	5	1		6	1	5	6	

SNP, single-nucleotide polymorphism.

a I, II, III, and IV represent the presence of the subgenome-specific SNP at ≤5 bp, 6−10 bp, 11−15 bp, and ≥16 bp from the 3′ end of the assay primer, respectively. Null represents no subgenome-specific SNP.

Because most current available SNP genotyping platforms prefer that there be no additional varietal SNP(s) within the 20-bp flanking regions of the targeted nucleotide being assayed, we separated our transcriptome-derived varietal SNPs into three types: type I (15,991), with no additional varietal SNP(s) within the 100-bp regions either side of a varietal SNP; type II (6130), with one or more varietal SNPs within the 21-100-bp flanking regions of a varietal SNP; and type III (15,292), with additional varietal SNP(s) within the 20-bp flanking regions of a varietal SNP (File S1). Given a large enough number of SNPs to choose from, it should be possible to bias the selection of SNPs to those that will behave as co-dominant assays by choosing those that have flanking genome-specific SNPs within 20 bp.

### Construction of genetic linkage map and QTL mapping

**Figure 6:**
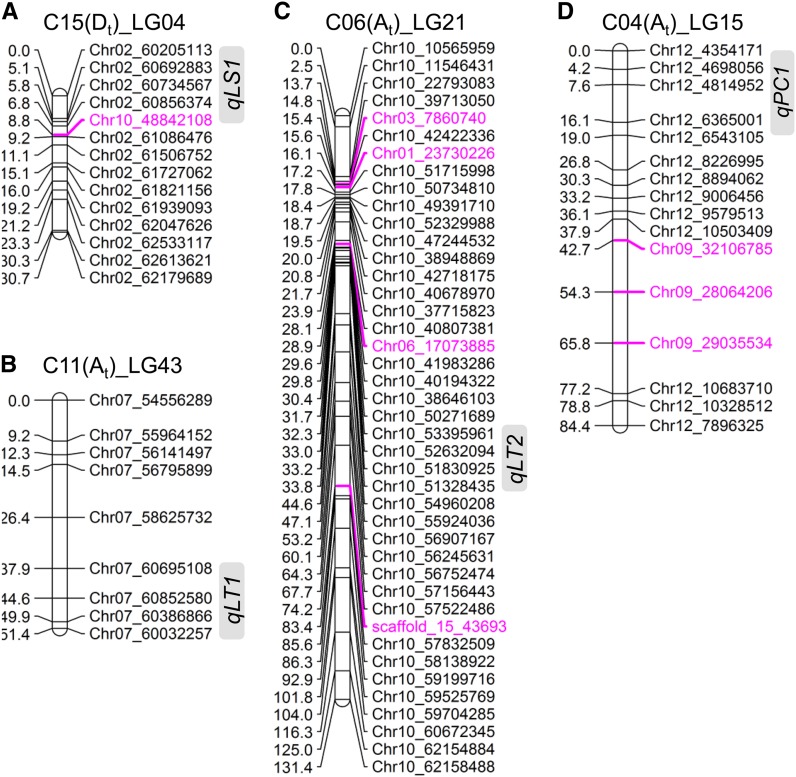
Linkage groups with mapped QTL. For each chromosome, only the linkage group (LG) with a QTL mapped was shown. Each linkage group was named by the chromosome number of *G. hirsutum*, the corresponding subgenome (A_t_ or D_t_), and LG in this study. SNPs were named based on the chromosome number of *G. raimondii* followed by their alignment coordinates on the corresponding *G. raimondii* chromosome. SNP marker(s) with its origin different from the majority SNPs that defined the linkage group are shown in pink. The QTL regions were indicated by gray rectangles.

A *G. hirsutum* genetic linkage map with 1244 SNP markers (not including 25 redundant SNP markers) was constructed based on an F_7_ RIL population derived from MCU-5 × Siokra 1−4 (File S3). This map contains 54 linkage groups with a total genetic distance of 5557.42 cM. These linkage groups were first assigned to a corresponding *G. raimondii* chromosome based on the localization of the majority of SNPs within the linkage group and then assigned to A_t_ or D_t_ subgenome based on the origin (A_2_ or D_5_) of the majority of the RNA-seq derived SNPs of each linkage group. This was determined by comparison of SNP containing reads with the transcriptome data from the A_2_ genome (*G. arboretum*; our own unpublished data) and the published *G. raimondii* (D_5_) genome sequence (Paterson *et al.* 2012). Of the 914 RNA-seq derived SNPs, for which a subgenome (A_t_ or D_t_) origin was bioinformatically determined, 905 (99%) were correctly mapped to a corresponding A_t_ or D_t_ linkage group. Of the 1244 SNP markers, 526 (42.28%) and 718 (57.72%) were mapped to the A_t_ and D_t_ linkage groups, respectively. Each of the 26 chromosomes of *G. hirsutum* was represented by 1−4 linkage groups. Generally, colinearity of the SNP markers between *G. raimondii* and *G. hirsutum* was observed for the majority of linkage groups although minor intrachromosomal inversions existed in some of the linkage groups. In total, 80 SNP markers (6.43%) did not group with their corresponding chromosome (those highlighted in pink in Figure 6 and File S3). For example, SNP marker Chr10_48842108 was mapped to *G. raimondii* Chr02 [C15(D_t_)_LG04], and SNP markers Chr09_32106785, Chr09_28064206, and Chr09_29035534 were mapped to *G. raimondii* Chr12 [C04(A_t_)_LG15] (Figure 6, A and D). Some of these could be artifacts of the mapping, but some could be due to chromosome rearrangement. For example, a number of SNP markers from *G. raimondii* Chr03 were always grouped with those from *G. raimondii* Chr05 (File S3), which is a result of chromosome arm translocation between C02(A_t_) (*G. raimondii* Chr05) and C03(A_t_) (*G. raimondii* Chr03) in *G. hirsutum* (Rong *et al.* 2004; Wang *et al.* 2013). Another potential chromosome arm translocation in *G. hirsutum* was between C04(A_t_) and C05(A_t_) (Rong *et al.* 2004; Wang *et al.* 2013). We found that this translocation could in fact be between their counterparts in the D_t_ subgenome, *i.e.*, between C19 (*G. raimondii* Chr09) and C22 (*G. raimondii* Chr12) because all RNA-seq derived SNP markers mapped to *G. raimondii* Chr09 and Chr12 in LG16 were from the D_t_ subgenome (File S3).

Leaf shape in cotton affects yield, insect resistance, lint trash, and the efficacy of foliar chemical application (Andres *et al.* 2014). Cotton leaf trichomes serve various functions, including protection against herbivores, insect pests, and drought (Desai *et al.* 2008). These two traits have been extensively investigated and QTL related to leaf shape and trichome have been reported in cotton (Wright *et al.* 1999; Jiang *et al.* 2000; Lacape and Nguyen 2005; Desai *et al.* 2008; Lacape *et al.* 2013; Andres *et al.* 2014). In this study, we chose these two traits together with another morphologic marker trait, pollen color, in QTL analyses to assess the usefulness of the SNP marker-only linkage map in QTL mapping (Figure 6). A major leaf shape QTL (*qLS1*, 62% phenotypic contribution) was mapped to an 8.8-cM interval in linkage group C15(D_t_)_LG04, consistent with previous results achieved in other populations (Jiang *et al.* 2000; Lacape *et al.* 2013; Andres *et al.* 2014). Two major leaf trichome density QTL (*qLT1* and *qLT2*, 31% and 28% phenotypic contribution, respectively) were identified in linkage groups ^11^C(A_t_)_LG43 and C06(A_t_)_LG21. *qLT2* has been previously reported in other population (Wright *et al.* 1999; Lacape and Nguyen 2005; Desai *et al.* 2008) whereas *qLT1* is a newly identified QTL in the MCU-5 × Siokra 1−4 population. *qPC1*, a major QTL (91% phenotypic contribution) for pollen color, was mapped to a 19-cM interval in linkage group C04(A_t_)_LG15. In a recent report, several QTL for pollen color were mapped to C05 (A_t_ subgenome) in a region centered on SSR marker CIR253 (Lacape *et al.* 2013), which according to Wang *et al.* (2013) was mapped to *G. raimondii* Chr12 (C04, between 4554023 bp and 4554387 bp) rather than C05 (*G. raimondii* Chr09), consistent with our result.

## Discussion

In this study, we showed that the rationale we proposed worked well for the identification of genuine varietal or allelic SNPs in tetraploid cotton as opposed to the mostly subgenome-specific SNPs that have dominated many earlier SNP discovery projects in cotton based on the analysis of cotton ESTs in database collections. The validity of our approach was demonstrated by the high verification rate (72.6–91.7%) obtained with a reasonably large number of the identified varietal SNPs (~1700) and several different SNP genotyping platforms (Table 3). Our analyses generated a large number of potential varietal SNPs (~38 K) across a range of commercial varieties that form the base for much of the diversity in commercial cotton breeding in Australia and elsewhere. The international deployment of Australian varieties under the FiberMax brand over the last decade, and their use in a number of major US breeding programs means that the SNPs will have widespread utility and a significant proportion of them have been made available as part of an international cotton community SNP chip now marketed by Illumina (http://www.illumina.com/applications/agriculture/consortia.ilmn).

To date, only two other published studies have reported large-scale varietal SNP identification in *G. hirsutum*, and both used NGS and the complexity-reduced DNA sequencing approach (Byers *et al.* 2012; Rai *et al.* 2013), but neither had the potential breadth of utility for applications to *G. hirsutum* breeding as those reported here. Rai *et al.* (2013) identified a large number of SNPs among six Indian *G. hirsutum* accessions, but only a small number (30 SNPs) were selected for validation and it is still unclear what proportion are reliable allelic SNPs rather than just subgenome-specific SNPs. Further verification using more SNPs and a broader range of germplasm is required to establish the reliability of those predicted SNPs. In the second study, where SNPs between a wild and a cultivated cotton were determined, less than 40% of the 1052 SNPs genotyped were found to amplify or segregate in an expected ratio in an F_2_ population derived from the two *G. hirsutum* accessions used in SNP identification (Byers *et al.* 2012), so validation rates were low. One of the accessions is a wild race cotton and at the extreme edge of the diversity range in tetraploid *G. hirsutum*, so even the validated SNPs would not expected to be polymorphic among commercial varieties. In other polyploids, despite a verification rate of 93% being recently reported in *Brassica napus* (Huang *et al.* 2013), most had a verification rate of less than 70% (Allen *et al.* 2011; Trebbi *et al.* 2011). Therefore, this study not only provided the cotton community a significant number of high-quality *G. hirsutum* SNPs but has contributed a novel way for identification of highly confident varietal SNPs between tetraploid varieties.

Two other strategies have previously been used to identify cotton varietal SNPs from NGS data, although neither has proven to be very satisfactory. One was to *de novo* assemble high-throughput sequencing reads together from two varieties at a time and then use the automated calling function in AutoSNP (Barker *et al.* 2003) to detect varietal SNPs and exclude subgenome-specific SNPs (Rai *et al.* 2013). Accurate SNP identification with AutoSNP depends on coalignment of reads from homeologous genes, but avoiding coalignment of reads from paralogous genes. According to Salmon *et al.* (2010), a 97% identity rate or read mapping stringency was able to separate putative paralogs in cotton and this was the stringency cutoff used by Rai *et al.* (2013). However, given the sequence depth cutoff (at least three reads per genotype) used by those authors and consequent lack of discrimination between subgenome reads, it is likely that many of their putative varietal SNPs are subgenome-specific SNPs where, by chance or differential expression of homeologs, not all the alleles in one variety were successfully sampled during sequencing (as in a scenario like that shown in Figure 1A, when only A_t_ or D_t_ reads were present for variety 2). A second strategy was to separately align the A_t_ and D_t_ genome reads from two varieties using very stringent alignment parameters or a precharacterized genome-specific SNP index to separate short reads into their subgenome of origin before varietal SNP identification (Byers *et al.* 2012; Salmon *et al.* 2012; Page *et al.* 2013a,b). This is based on the assumption that SNPs in the ancestral diploids (A_2_: *G. arboreum* and D_5_: *G. raimondii*) have remained unaltered since polyploidy formation and can be used as diagnostic subgenome-specific SNPs in modern tetraploid cotton (Salmon *et al.* 2010). Varietal SNPs can then be separately identified within each subgenome-specific assembly using the approaches and tools used for diploids, such as SAMtools (Li *et al.* 2009). However, it was found that ~30% of reads from allopolyploid cotton that mapped to the *G. raimondii* genome were indistinguishable between the A_t_ and D_t_ genome (Page *et al.* 2013b); therefore, these reads cannot be separated and will still be coaligned.

In addition, although ~76% of the subgenome-specific SNPs found in modern tetraploid cotton were present in the ancestral A and D genomes, the remainder were newly evolved after polyploidization (Page *et al.* 2013b), so short reads containing these newly evolved subgenome-specific SNPs cannot be separated using the precharacterized, genome-specific SNP index developed based on the ancestral diploid genomes. Our approach, on the other hand, takes advantage of the *G. raimondii* (D_5_) genome sequence (Paterson *et al.* 2012; Wang *et al.* 2012b) and used it as the scaffold on which to align the RNA-seq reads allowing sufficient mismatches to capture homeologs but not paralogs. Since the short read sequences are transcript derived, differential expression between homeologs within varieties must also be considered because this may affect sampling of different alleles in the sequence space. Biased expression of A_t_ and D_t_ homeologs has been well documented in several studies in cotton (Adams *et al.* 2003; Rapp *et al.* 2009; Flagel and Wendel 2010; Yoo *et al.* 2013), and at least 40% of homeologs were reported to be transcriptionally biased in at least one stage of cotton development (Chaudhary *et al.* 2009). To address this issue, we used the presence of subgenome-specific SNPs to filter alignments to those known to have both A_t_ and D_t_ reads represented from each pair of varieties and thence to confidently call adjacent varietal SNPs within the co-aligned reads in a 100-bp sliding window. Our approach avoided the problem associated with the AutoSNP approach, *i.e.*, false SNP calls caused by differential or biased allelic expression, and adopted the advantage of the separate subgenome alignment approach, *i.e.*, use of subgenome-specific SNPs to distinguish reads from the two subgenomes, which makes it possible to more confidently call a potential varietal SNP (Table 3).

More importantly, our approach did not specifically need to distinguish which allele is from the A_t_ genome and which allele is from the D_t_ genome, just that the SNP was biallelic in both genotypes being compared (although in many cases that should be possible with reference to the genomic resources now available for A_2_- and D_5_-genome cottons). This makes our approach more adaptable in polyploids without reference genome sequences. The constraints we have imposed through our strategy would necessarily underestimate the number of varietal SNPs between genotypes because our approach i) cannot identify varietal SNPs located in regions unique to either the A_t_ or D_t_ genome, as our SNP calling relies on the presence of both A_t_ and D_t_ reads; and ii) will miss the genuine varietal SNPs without a flanking subgenome-specific SNP. Nevertheless, in terms of ease of automation and reliability of prediction, our approach is a considerable improvement in SNP detection in the absence of a full tetraploid genome sequence and even when that sequence is available, will still offer some advantages over other automated approaches.

Complexity-reduced DNA-sequencing strategies, such as RAD (Baird *et al.* 2008) and GBS (Elshire *et al.* 2011; Poland *et al.* 2012), have been applied in many species to identify SNPs, particularly in those without a reference genome (Barchi *et al.* 2011; Yang *et al.* 2012; Saintenac *et al.* 2013). A number of bioinformatic tools, such as Stacks (Catchen *et al.* 2011) and UNEAK (Lu *et al.* 2013), have been developed to handle this type of sequencing data for SNP discovery and genotyping. In this study, we used the publically available “off-the-shelf” windows-based tool, the CLC Genomics Workbench, which is accessible to wet-lab biologists, in SNP identification in tetraploid cotton. The software is designed to identify sequence variations between one accession and its reference sequence rather than sequence variations between two varieties but was adapted to this purpose through some preprocessing and filtering of the read data. By changing the format of the input data (sequence tags instead of sequence reads), setting up proper mapping and filtering parameters followed by manual checking, we successfully identified large numbers of varietal SNPs with a high verification rate in two tetraploid cotton varieties using the CLC Genomics Workbench (Table 3), contributing an alternative way for SNP identification, although again relying on adjacent subgenome-specific SNPs to increase the confidence of the calls. This approach, however, is only applicable for RAD or GBS sequencing data and not for RNA-seq data because each RNA-seq read is unique and cannot be collapsed into sequence tags in the same way as RAD or GBS reads.

When allowing multiple aligned positions, We found that ~70% of the *G. hirsutum* transcriptome reads could be aligned to the *G. raimondii* genome, suggesting that the remaining reads could derive from genes either unique to the A_t_ genome, or that have diverged significantly from those of their common ancestor with *G. raimondii*. When allowing only a single best aligned position (≤4 mismatches), 37–50% of *G. hirsutum* transcriptome reads could still be aligned to *G. raimondii* (Table 1). In contrast, only about one third of the RAD reads could be aligned to the *G. raimondii* genome, even allowing up to eight mismatches. These results suggest that, in terms of read alignment and SNP identification, the *G. raimondii* reference works well for transcriptome derived reads but may not be able to fully support SNP identification from short reads generated from complexity-reduced DNA sequencing, either because of divergence between the D_t_ and D_5_ genomes or because the reference is just less reliable in those regions where the assembly is not supported by complementary EST or transcriptome data. Alignments will hopefully be improved by using the newly released A_2_-genome sequence or when a high-quality tetraploid cotton genome sequence becomes available, by which stage most cotton marker discovery and assessments will be through direct GBS.

A codominant SNP assay will be more useful in genetic mapping and breeding programs that frequently deal with segregating populations, such as F_2_ and backcross populations. In our SNP datasets, only ~3% were predicted to behave as codominant assays, which was significantly lower than that in wheat, where 10–20% of SNPs identified were simple codominant SNPs (Allen *et al.* 2013). This could be related to our stringent criterion used in SNP identification but the possibility that a low frequency of such “co-dominant” type SNP markers naturally occurs in cotton could not be ruled out. However, in practice, we found that a potentially dominant acting SNP assay could act as a codominant SNP assay when one or more genome-specific SNP(s) are present in the assay primers such that they amplify only the allele in which the varietal SNP resides. Genome-specific SNP(s) can be included in the allele-specific primers, the universal primer or both (Table 4). Where practical, it is better to include as many genome-specific SNP(s) as possible and to have them in both primers to ensure robust allele specificity. SNP assays designed to specifically amplify only the subgenome carrying the SNP has been attempted previously in cotton although with lower than expected success rates (Byers *et al.* 2012). Our genotyping data found more codominant SNP assays in the RAD-based SNPs than in the transcriptome-based SNPs, probably because noncoding sequences are more divergent and contain more genome-specific SNPs than coding sequences.

Cotton genetic maps so far used in QTL mapping were mainly constructed using non-SNP markers, mostly SSR markers, although a genetic map with 1104 markers, including 414 SNP markers, has recently been used in mapping of QTL resistant to Verticillum wilt (Fang *et al.* 2014) and a map with only SNP markers (346 in total) has been constructed (Byers *et al.* 2012). In this study we constructed a *G. hirsutum* genetic linkage map with 1244 of our identified SNP markers that were distributed across all 26 *G. hirsutum* chromosomes and also used them to map QTL for leaf shape, leaf trichome density, and pollen color on this SNP only map. Although three out of the four major QTL reported in this study were confirmations of earlier studies in different populations (Wright *et al.* 1999; Jiang *et al.* 2000; Lacape and Nguyen 2005, Desai *et al.* 2008; Lacape *et al.* 2013; Andres *et al.* 2014), one new QTL was found for leaf trichome density (Figure 6B). In addition, the QTL intervals identified in this study were defined by the positions of the SNPs (based on *G. raimondii*), and the genes annotated in the intervals can then be screened and investigated to single out potential candidate(s) contributing to the QTL. There are 87, 74, 87, and 151 annotated genes in the regions corresponding to *qLS1*, *qLT1*, *qLT2*, and *qPC1* in *G. raimondii*, respectively (File S4). Recently, the region corresponding to *qLS1* has been further narrowed down to containing only 34 annotated genes, from which Gorai.002G244000 and Gorai.002G244200 that encode HD-Zip transcription factors were suggested to be the possible candidates for the leaf shape trait (Andres *et al.* 2014). Further investigation is required to confirm this speculation; nevertheless, use of SNP markers with a position designated based on the genome sequence of *G. raimondii* should be able to speed up the procedure of QTL fine mapping and identification of candidate genes underlying QTL of interest. Our full set of markers will have great utility in mapping more complex traits in cotton such as disease resistance or fiber yield and quality that are often conferred by many genes of small effect and will advance the use of marker-assisted selection in cotton breeding.

Using transcriptome and complexity-reduced-DNA sequencing, we identified a large number of varietal SNPs among 18 *G. hirsutum* varieties based on a robust protocol that relied on adjacent subgenome-specific SNPs to increase the confidence of SNP assignment to single alleles. A verification rate of 72.6–91.7% was achieved and ~25,000 of these SNPs satisfy the criteria for use in a number of common SNP genotyping platforms. Our pool of SNPs span a range of commercial and elite germplasm of *G. hirsutum* and so provides valuable marker resource for the cotton community as demonstrated by mapping of QTL for several traits of interest. The SNP identification rationale described here should be applicable to other polyploids.

## Supplementary Material

Supporting Information
