# Supplementary material for: Transcriptome and Complexity-Reduced, DNA-Based Identification of Intraspecies Single-Nucleotide Polymorphisms in the Polyploid Gossypium hirsutum L
Source: G3 (Bethesda). 2014 Aug 7;4(10):1893–905. doi: 10.1534/g3.114.012542 (PMC4199696; doi:10.1534/g3.114.012542)
Supplement: Supporting Information [file supp_4_10_1893__index.html]

Transcriptome and Complexity-Reduced, DNA-Based Identification of Intraspecies Single-Nucleotide Polymorphisms in the Polyploid Gossypium hirsutum L. — Supporting Information 

# Transcriptome and Complexity-Reduced, DNA-Based Identification of Intraspecies Single-Nucleotide Polymorphisms in the Polyploid *Gossypium hirsutum* L.

## Supporting Information for Zhu *et al.*, 2014

**Files in this Data Supplement:**

- Table S1 - Adaptor sequences used in creation of RAD libraries. (PDF, 103 KB)
- File S3 - Genetic linkage map of *G. hirsutum*. A 5557.42 cM map with 1,244 SNP markers distributed across 54 linkage groups was constructed using an F7 RIL population derived from MCU-5 x Siokra 1-4. Each *G. hirsutum* chromosome was named by its chromosome number, the corresponding sub-genome (At or Dt) and LG in this study. SNP marker(s) with its origin different from the majority SNPs that defined the linkage group are shown in pink. (PDF, 185 KB)
- File S1 - Transcriptome based SNPs identified among 18 *G. hirsutum* varieties. (.xlsx, 2 MB)
- File S2 - Complexity-reduced-genomic DNA based SNPs between MCU-5 and Siokra 1-4. (.xlsx, 292 KB)
- File S4 - Annotated genes in the QTL interval in *G. raimondii*. (.xlsx, 43 KB)
