# Supplementary material for: Transcriptome and Complexity-Reduced, DNA-Based Identification of Intraspecies Single-Nucleotide Polymorphisms in the Polyploid Gossypium hirsutum L
Source: G3 (Bethesda). 2014 Aug 7;4(10):1893–905. doi: 10.1534/g3.114.012542 (PMC4199696; doi:10.1534/g3.114.012542)
Supplement: Supporting Information [file supp_g3.114.012542_TableS1.pdf]

Table S1 Adaptor sequences used in creation of RAD libraries

Restriction enzyme and adaptor	Strand	Sequence[b]
Adaptor 1		
MCU-5		
*Eco*RI	Up	5’-AATGATACGGCGACCACCGAGATCTACACTCTTTCCCTACACGACGCTCTTCCGATCTATCACG-3’
	Bottom	5’-Phos-AATTCGTGATAGATCGGAAGAGCGTCGTGTAGGGAAAGAGTGTAGATCTCGGTGGTCGCCGTATCATT-3’
*Ape*KI	Up	5’-AATGATACGGCGACCACCGAGATCTACACTCTTTCCCTACACGACGCTCTTCCGATCTATCACG-3’
	Bottom[a]	5’-Phos-CWGCGTGATAGATCGGAAGAGCGTCGTGTAGGGAAAGAGTGTAGATCTCGGTGGTCGCCGTATCATT-3’
*Sbf*I	Up	5’-AATGATACGGCGACCACCGAGATCTACACTCTTTCCCTACACGACGCTCTTCCGATCTATCACGTGCA-3
	Bottom	5’-Phos-CGTGATAGATCGGAAGAGCGTCGTGTAGGGAAAGAGTGTAGATCTCGGTGGTCGCCGTATCATT-3’
Siokra 1-4		
*Eco*RI	Up	5’-AATGATACGGCGACCACCGAGATCTACACTCTTTCCCTACACGACGCTCTTCCGATCTCGATGT-3’
	Bottom	5’-Phos-AATTACATCGAGATCGGAAGAGCGTCGTGTAGGGAAAGAGTGTAGATCTCGGTGGTCGCCGTATCATT-3’
*Ape*KI	Up	5’-AATGATACGGCGACCACCGAGATCTACACTCTTTCCCTACACGACGCTCTTCCGATCTCGATGT-3’
	Bottom[a]	5’-Phos-CWGACATCGAGATCGGAAGAGCGTCGTGTAGGGAAAGAGTGTAGATCTCGGTGGTCGCCGTATCATT-3’
*Sbf*I	Up	5’-AATGATACGGCGACCACCGAGATCTACACTCTTTCCCTACACGACGCTCTTCCGATCTCGATGTTGCA-3
	Bottom	5’-Phos-ACATCGAGATCGGAAGAGCGTCGTGTAGGGAAAGAGTGTAGATCTCGGTGGTCGCCGTATCATT-3’
Adaptor 2		
	Up	5’-Phos-CTCAGGCATCACTCGATTCCTCCGAGAACAA-3’
	Bottom	5’-CAAGCAGAAGACGGCATACGACGGAGGAATCGAGTGATGCCTGAGT-3’

[a] W=A or T; [b] index sequences are underlined.
