# Supplementary material for: Transcriptome and Complexity-Reduced, DNA-Based Identification of Intraspecies Single-Nucleotide Polymorphisms in the Polyploid Gossypium hirsutum L
Source: G3 (Bethesda). 2014 Aug 7;4(10):1893–905. doi: 10.1534/g3.114.012542 (PMC4199696; doi:10.1534/g3.114.012542)
Supplement: Supporting Information [file supp_g3.114.012542_FileS3.pdf]

File S3
Genetic linkage map of *G. hirsutum*.

A 5557.42 cM map with 1,244 SNP markers distributed across 54 linkage groups was constructed using an F_7 RIL population derived from MCU-5 x Siokra 1-4. Each *G. hirsutum* chromosome was named by its chromosome number, the corresponding sub-genome (A_t or D_t) and LG in this study. SNP marker(s) with its origin different from the majority SNPs that defined the linkage group are shown in pink.

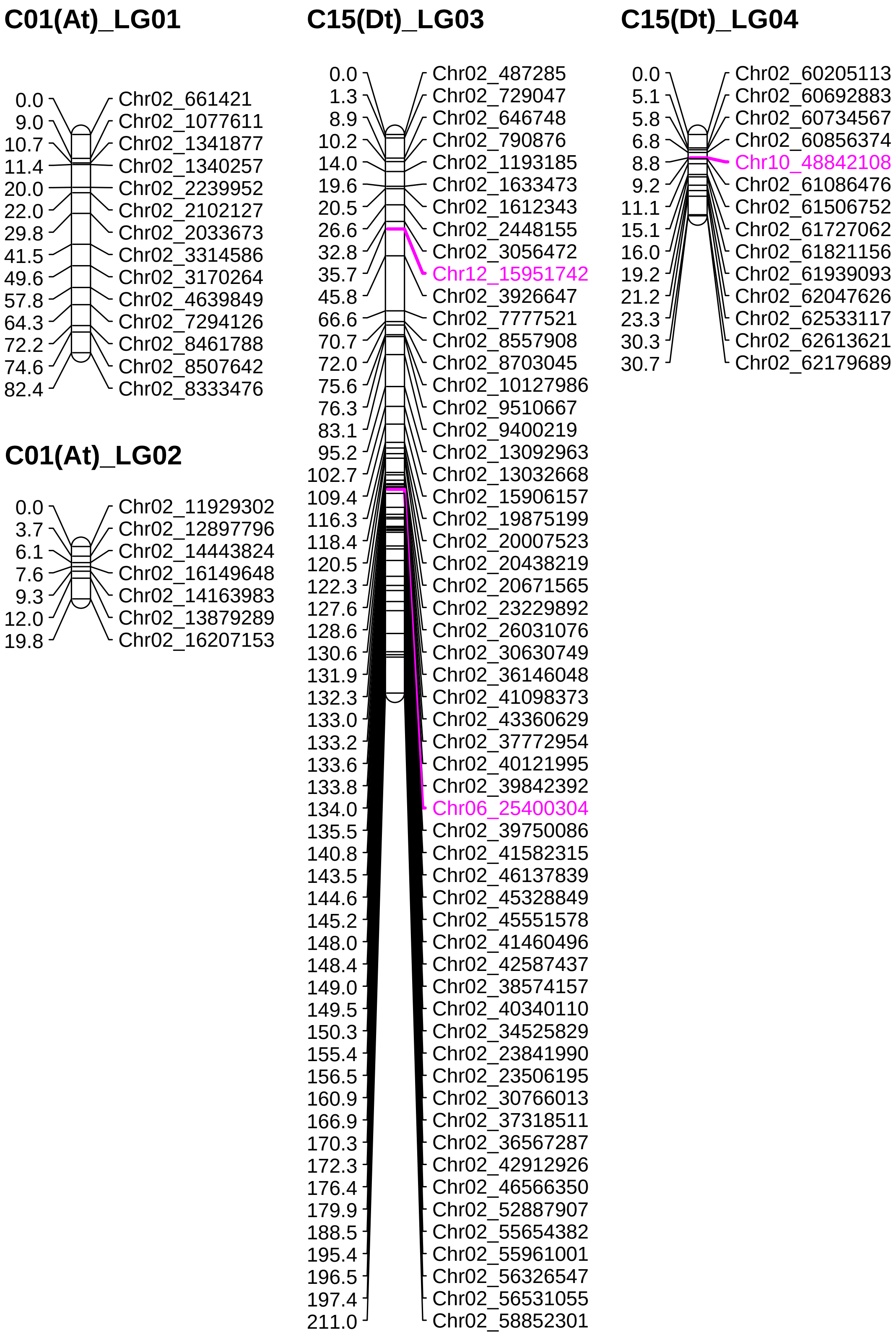
C01(At)_LG01
0.0 Chr02_661421
9.0 Chr02_1077611
10.7 Chr02_1341877
11.4 Chr02_1340257
20.0 Chr02_2239952
22.0 Chr02_2102127
29.8 Chr02_2033673
41.5 Chr02_3314586
49.6 Chr02_3170264
57.8 Chr02_4639849
64.3 Chr02_7294126
72.2 Chr02_8461788
74.6 Chr02_8507642
82.4 Chr02_8333476
C01(At)_LG02
0.0 Chr02_11929302
3.7 Chr02_12897796
6.1 Chr02_14443824
7.6 Chr02_16149648
9.3 Chr02_14163983
12.0 Chr02_13879289
19.8 Chr02_16207153
C15(Dt)_LG03
0.0 Chr02_487285
1.3 Chr02_729047
8.9 Chr02_646748
10.2 Chr02_790876
14.0 Chr02_1193185
19.6 Chr02_1633473
20.5 Chr02_1612343
26.6 Chr02_2448155
32.8 Chr02_3056472
35.7 Chr12_15951742
45.8 Chr02_3926647
66.6 Chr02_7777521
70.7 Chr02_8557908
72.0 Chr02_8703045
75.6 Chr02_10127986
76.3 Chr02_9510667
83.1 Chr02_9400219
95.2 Chr02_13092963
102.7 Chr02_13032668
109.4 Chr02_15906157
116.3 Chr02_19875199
118.4 Chr02_20007523
120.5 Chr02_20438219
122.3 Chr02_20671565
127.6 Chr02_23229892
128.6 Chr02_26031076
130.6 Chr02_30630749
131.9 Chr02_36146048
132.3 Chr02_41098373
133.0 Chr02_43360629
133.2 Chr02_37772954
133.6 Chr02_40121995
133.8 Chr02_39842392
134.0 Chr06_25400304
135.5 Chr02_39750086
140.8 Chr02_41582315
143.5 Chr02_46137839
144.6 Chr02_45328849
145.2 Chr02_45551578
148.0 Chr02_41460496
148.4 Chr02_42587437
149.0 Chr02_38574157
149.5 Chr02_40340110
150.3 Chr02_34525829
155.4 Chr02_23841990
156.5 Chr02_23506195
160.9 Chr02_30766013
166.9 Chr02_37318511
170.3 Chr02_36567287
172.3 Chr02_42912926
176.4 Chr02_46566350
179.9 Chr02_52887907
188.5 Chr02_55654382
195.4 Chr02_55961001
196.5 Chr02_56326547
197.4 Chr02_56531055
211.0 Chr02_58852301
C15(Dt)_LG04
0.0 Chr02_60205113
5.1 Chr02_60692883
5.8 Chr02_60734567
6.8 Chr02_60856374
8.8 Chr10_48842108
9.2 Chr02_61086476
11.1 Chr02_61506752
15.1 Chr02_61727062
16.0 Chr02_61821156
19.2 Chr02_61939093
21.2 Chr02_62047626
23.3 Chr02_62533117
30.3 Chr02_62613621
30.7 Chr02_62179689

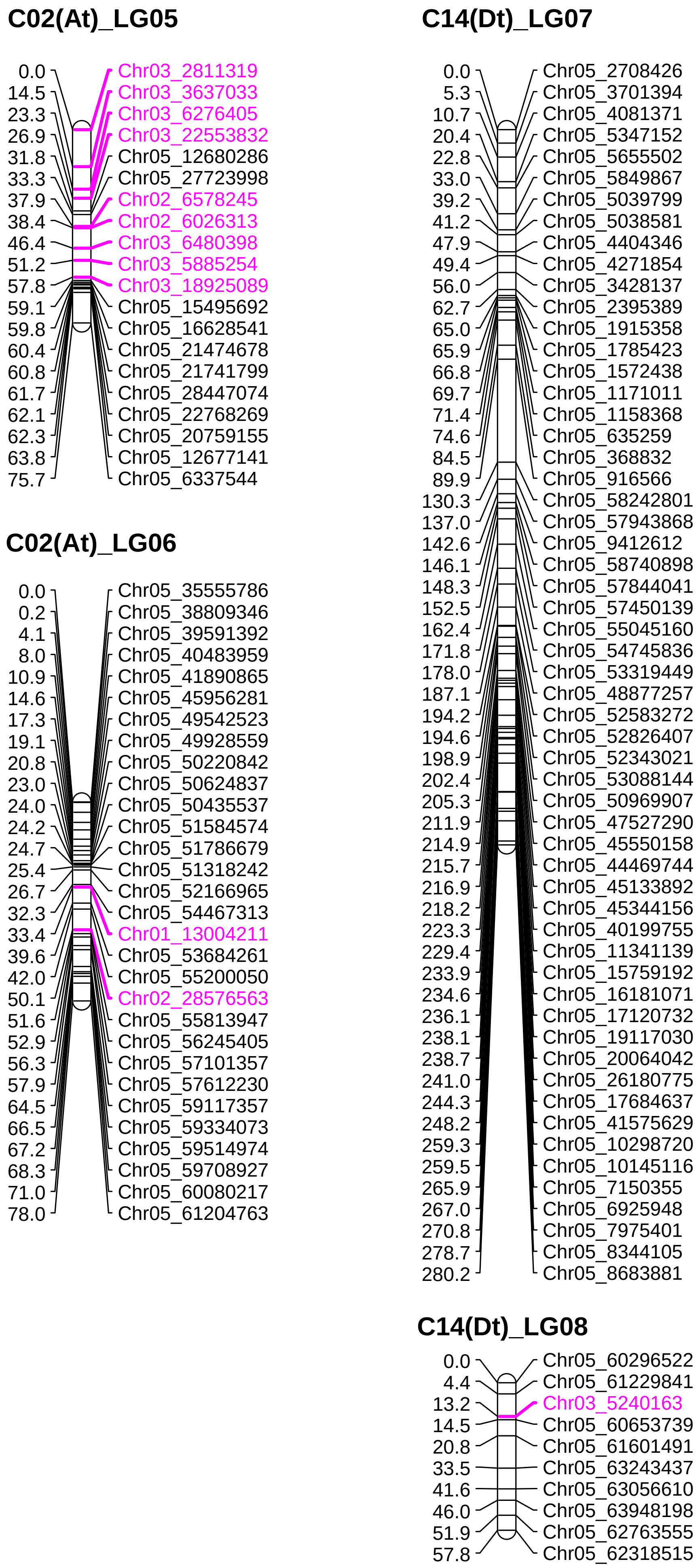
C02(At)_LG05
0.0 Chr03_2811319
14.5 Chr03_3637033
23.3 Chr03_6276405
26.9 Chr03_22553832
31.8 Chr05_12680286
33.3 Chr05_27723998
37.9 Chr02_6578245
38.4 Chr02_6026313
46.4 Chr03_6480398
51.2 Chr03_5885254
57.8 Chr03_18925089
59.1 Chr05_15495692
59.8 Chr05_16628541
60.4 Chr05_21474678
60.8 Chr05_21741799
61.7 Chr05_28447074
62.1 Chr05_22768269
62.3 Chr05_20759155
63.8 Chr05_12677141
75.7 Chr05_6337544
C02(At)_LG06
0.0 Chr05_35555786
0.2 Chr05_38809346
4.1 Chr05_39591392
8.0 Chr05_40483959
10.9 Chr05_41890865
14.6 Chr05_45956281
17.3 Chr05_49542523
19.1 Chr05_49928559
20.8 Chr05_50220842
23.0 Chr05_50624837
24.0 Chr05_50435537
24.2 Chr05_51584574
24.7 Chr05_51786679
25.4 Chr05_51318242
26.7 Chr05_52166965
32.3 Chr05_54467313
33.4 Chr01_13004211
39.6 Chr05_53684261
42.0 Chr05_55200050
50.1 Chr02_28576563
51.6 Chr05_55813947
52.9 Chr05_56245405
56.3 Chr05_57101357
57.9 Chr05_57612230
64.5 Chr05_59117357
66.5 Chr05_59334073
67.2 Chr05_59514974
68.3 Chr05_59708927
71.0 Chr05_60080217
78.0 Chr05_61204763
C14(Dt)_LG07
0.0 Chr05_2708426
5.3 Chr05_3701394
10.7 Chr05_4081371
20.4 Chr05_5347152
22.8 Chr05_5655502
33.0 Chr05_5849867
39.2 Chr05_5039799
41.2 Chr05_5038581
47.9 Chr05_4404346
49.4 Chr05_4271854
56.0 Chr05_3428137
62.7 Chr05_2395389
65.0 Chr05_1915358
65.9 Chr05_1785423
66.8 Chr05_1572438
69.7 Chr05_1171011
71.4 Chr05_1158368
74.6 Chr05_635259
84.5 Chr05_368832
89.9 Chr05_916566
130.3 Chr05_58242801
137.0 Chr05_57943868
142.6 Chr05_9412612
146.1 Chr05_58740898
148.3 Chr05_57844041
152.5 Chr05_57450139
162.4 Chr05_55045160
171.8 Chr05_54745836
178.0 Chr05_53319449
187.1 Chr05_48877257
194.2 Chr05_52583272
194.6 Chr05_52826407
198.9 Chr05_52343021
202.4 Chr05_53088144
205.3 Chr05_50969907
211.9 Chr05_47527290
214.9 Chr05_45550158
215.7 Chr05_44469744
216.9 Chr05_45133892
218.2 Chr05_45344156
223.3 Chr05_40199755
229.4 Chr05_11341139
233.9 Chr05_15759192
234.6 Chr05_16181071
236.1 Chr05_17120732
238.1 Chr05_19117030
238.7 Chr05_20064042
241.0 Chr05_26180775
244.3 Chr05_17684637
248.2 Chr05_41575629
259.3 Chr05_10298720
259.5 Chr05_10145116
265.9 Chr05_7150355
267.0 Chr05_6925948
270.8 Chr05_7975401
278.7 Chr05_8344105
280.2 Chr05_8683881
C14(Dt)_LG08
0.0 Chr05_60296522
4.4 Chr05_61229841
13.2 Chr03_5240163
14.5 Chr05_60653739
20.8 Chr05_61601491
33.5 Chr05_63243437
41.6 Chr05_63056610
46.0 Chr05_63948198
51.9 Chr05_62763555
57.8 Chr05_62318515

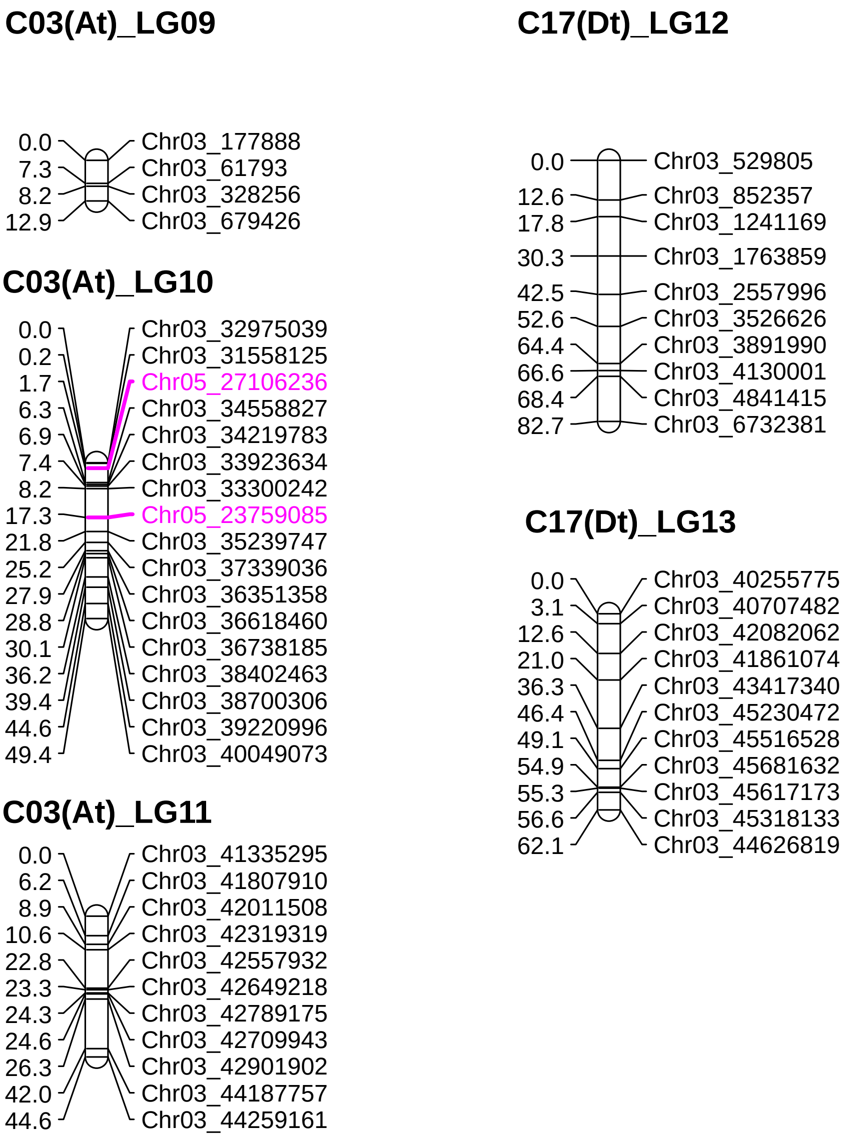

C03(At)_LG09
0.0 Chr03_177888
7.3 Chr03_61793
8.2 Chr03_328256
12.9 Chr03_679426
C03(At)_LG10
0.0 Chr03_32975039
0.2 Chr03_31558125
1.7 Chr05_27106236
6.3 Chr03_34558827
6.9 Chr03_34219783
7.4 Chr03_33923634
8.2 Chr03_33300242
17.3 Chr05_23759085
21.8 Chr03_35239747
25.2 Chr03_37339036
27.9 Chr03_36351358
28.8 Chr03_36618460
30.1 Chr03_36738185
36.2 Chr03_38402463
39.4 Chr03_38700306
44.6 Chr03_39220996
49.4 Chr03_40049073
C03(At)_LG11
0.0 Chr03_41335295
6.2 Chr03_41807910
8.9 Chr03_42011508
10.6 Chr03_42319319
22.8 Chr03_42557932
23.3 Chr03_42649218
24.3 Chr03_42789175
24.6 Chr03_42709943
26.3 Chr03_42901902
42.0 Chr03_44187757
44.6 Chr03_44259161
C17(Dt)_LG12
0.0 Chr03_529805
12.6 Chr03_852357
17.8 Chr03_1241169
30.3 Chr03_1763859
42.5 Chr03_2557996
52.6 Chr03_3526626
64.4 Chr03_3891990
66.6 Chr03_4130001
68.4 Chr03_4841415
82.7 Chr03_6732381
C17(Dt)_LG13
0.0 Chr03_40255775
3.1 Chr03_40707482
12.6 Chr03_42082062
21.0 Chr03_41861074
36.3 Chr03_43417340
46.4 Chr03_45230472
49.1 Chr03_45516528
54.9 Chr03_45681632
55.3 Chr03_45617173
56.6 Chr03_45318133
62.1 Chr03_44626819

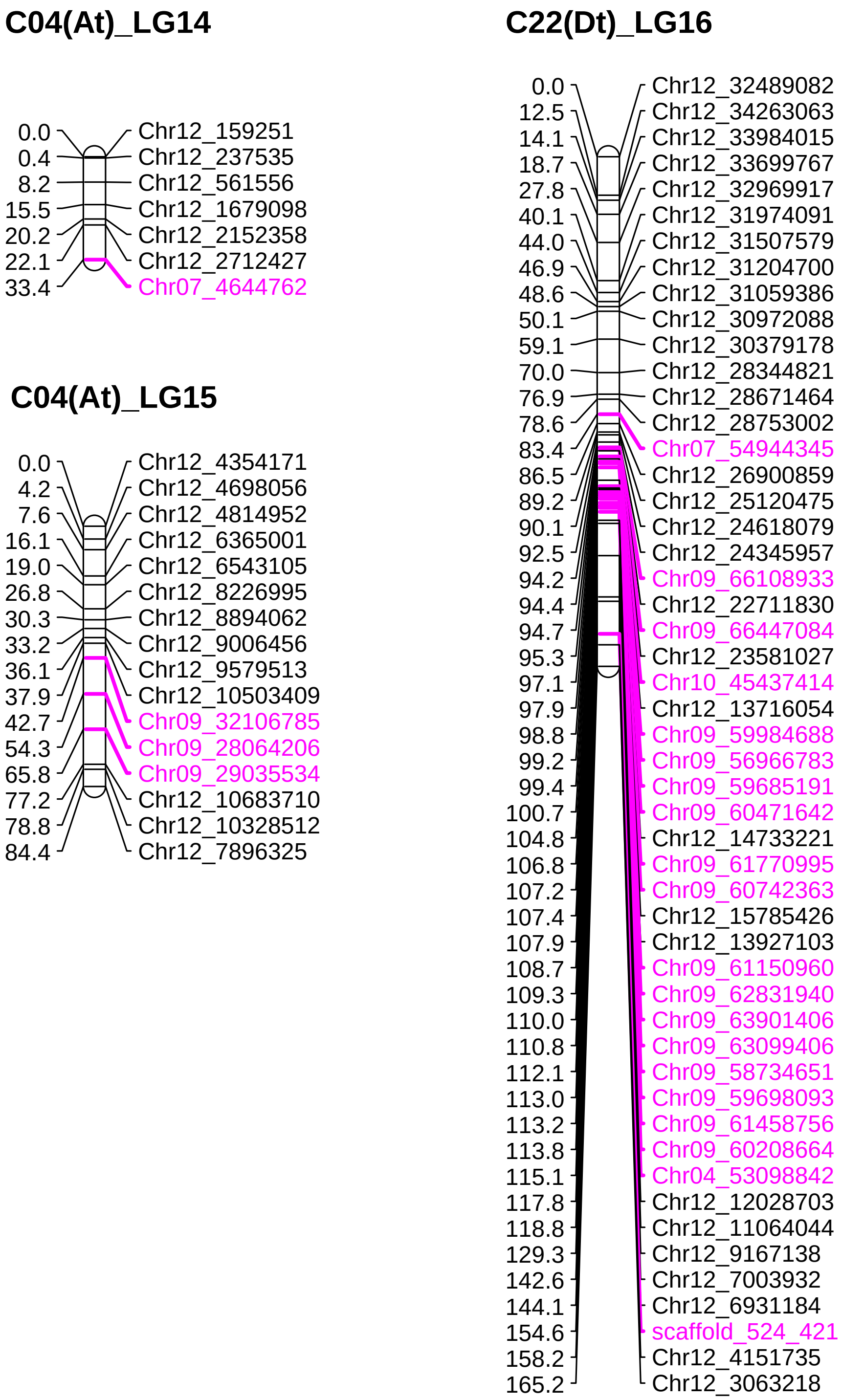
C04(At)_LG14
0.0 Chr12_159251
0.4 Chr12_237535
8.2 Chr12_561556
15.5 Chr12_1679098
20.2 Chr12_2152358
22.1 Chr12_2712427
33.4 Chr07_4644762
C04(At)_LG15
0.0 Chr12_4354171
4.2 Chr12_4698056
7.6 Chr12_4814952
16.1 Chr12_6365001
19.0 Chr12_6543105
26.8 Chr12_8226995
30.3 Chr12_8894062
33.2 Chr12_9006456
36.1 Chr12_9579513
37.9 Chr12_10503409
42.7 Chr09_32106785
54.3 Chr09_28064206
65.8 Chr09_29035534
77.2 Chr12_10683710
78.8 Chr12_10328512
84.4 Chr12_7896325
C22(Dt)_LG16
0.0 Chr12_32489082
12.5 Chr12_34263063
14.1 Chr12_33984015
18.7 Chr12_33699767
27.8 Chr12_32969917
40.1 Chr12_31974091
44.0 Chr12_31507579
46.9 Chr12_31204700
48.6 Chr12_31059386
50.1 Chr12_30972088
59.1 Chr12_30379178
70.0 Chr12_28344821
76.9 Chr12_28671464
78.6 Chr12_28753002
83.4 Chr07_54944345
86.5 Chr12_26900859
89.2 Chr12_25120475
90.1 Chr12_24618079
92.5 Chr12_24345957
94.2 Chr09_66108933
94.4 Chr12_22711830
94.7 Chr09_66447084
95.3 Chr12_23581027
97.1 Chr10_45437414
97.9 Chr12_13716054
98.8 Chr09_59984688
99.2 Chr09_56966783
99.4 Chr09_59685191
100.7 Chr09_60471642
104.8 Chr12_14733221
106.8 Chr09_61770995
107.2 Chr09_60742363
107.4 Chr12_15785426
107.9 Chr12_13927103
108.7 Chr09_61150960
109.3 Chr09_62831940
110.0 Chr09_63901406
110.8 Chr09_63099406
112.1 Chr09_58734651
113.0 Chr09_59698093
113.2 Chr09_61458756
113.8 Chr09_60208664
115.1 Chr04_53098842
117.8 Chr12_12028703
118.8 Chr12_11064044
129.3 Chr12_9167138
142.6 Chr12_7003932
144.1 Chr12_6931184
154.6 scaffold_524_421
158.2 Chr12_4151735
165.2 Chr12_3063218

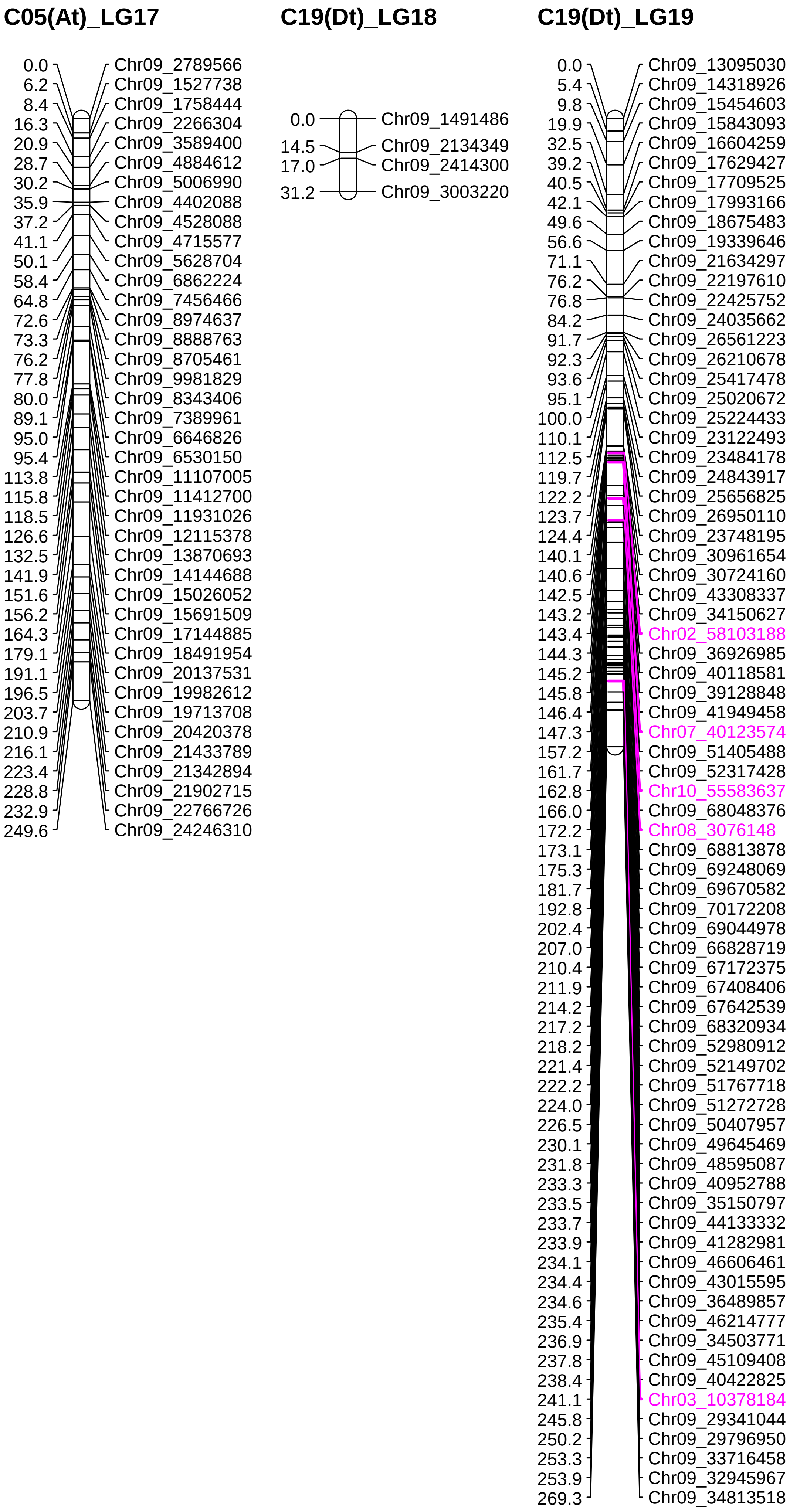

C05(At)_LG17
0.0 Chr09_2789566
6.2 Chr09_1527738
8.4 Chr09_1758444
16.3 Chr09_2266304
20.9 Chr09_3589400
28.7 Chr09_4884612
30.2 Chr09_5006990
35.9 Chr09_4402088
37.2 Chr09_4528088
41.1 Chr09_4715577
50.1 Chr09_5628704
58.4 Chr09_6862224
64.8 Chr09_7456466
72.6 Chr09_8974637
73.3 Chr09_8888763
76.2 Chr09_8705461
77.8 Chr09_9981829
80.0 Chr09_8343406
89.1 Chr09_7389961
95.0 Chr09_6646826
95.4 Chr09_6530150
113.8 Chr09_11107005
115.8 Chr09_11412700
118.5 Chr09_11931026
126.6 Chr09_12115378
132.5 Chr09_13870693
141.9 Chr09_14144688
151.6 Chr09_15026052
156.2 Chr09_15691509
164.3 Chr09_17144885
179.1 Chr09_18491954
191.1 Chr09_20137531
196.5 Chr09_19982612
203.7 Chr09_19713708
210.9 Chr09_20420378
216.1 Chr09_21433789
223.4 Chr09_21342894
228.8 Chr09_21902715
232.9 Chr09_22766726
249.6 Chr09_24246310
C19(Dt)_LG18
0.0 Chr09_1491486
14.5 Chr09_2134349
17.0 Chr09_2414300
31.2 Chr09_3003220
C19(Dt)_LG19
0.0 Chr09_13095030
5.4 Chr09_14318926
9.8 Chr09_15454603
19.9 Chr09_15843093
32.5 Chr09_16604259
39.2 Chr09_17629427
40.5 Chr09_17709525
42.1 Chr09_17993166
49.6 Chr09_18675483
56.6 Chr09_19339646
71.1 Chr09_21634297
76.2 Chr09_22197610
76.8 Chr09_22425752
84.2 Chr09_24035662
91.7 Chr09_26561223
92.3 Chr09_26210678
93.6 Chr09_25417478
95.1 Chr09_25020672
100.0 Chr09_25224433
110.1 Chr09_23122493
112.5 Chr09_23484178
119.7 Chr09_24843917
122.2 Chr09_25656825
123.7 Chr09_26950110
124.4 Chr09_23748195
140.1 Chr09_30961654
140.6 Chr09_30724160
142.5 Chr09_43308337
143.2 Chr09_34150627
143.4 Chr02_58103188
144.3 Chr09_36926985
145.2 Chr09_40118581
145.8 Chr09_39128848
146.4 Chr09_41949458
147.3 Chr07_40123574
157.2 Chr09_51405488
161.7 Chr09_52317428
162.8 Chr10_55583637
166.0 Chr09_68048376
172.2 Chr08_3076148
173.1 Chr09_68813878
175.3 Chr09_69248069
181.7 Chr09_69670582
192.8 Chr09_70172208
202.4 Chr09_69044978
207.0 Chr09_66828719
210.4 Chr09_67172375
211.9 Chr09_67408406
214.2 Chr09_67642539
217.2 Chr09_68320934
218.2 Chr09_52980912
221.4 Chr09_52149702
222.2 Chr09_51767718
224.0 Chr09_51272728
226.5 Chr09_50407957
230.1 Chr09_49645469
231.8 Chr09_48595087
233.3 Chr09_40952788
233.5 Chr09_35150797
233.7 Chr09_44133332
233.9 Chr09_41282981
234.1 Chr09_46606461
234.4 Chr09_43015595
234.6 Chr09_36489857
235.4 Chr09_46214777
236.9 Chr09_34503771
237.8 Chr09_45109408
238.4 Chr09_40422825
241.1 Chr03_10378184
245.8 Chr09_29341044
250.2 Chr09_29796950
253.3 Chr09_33716458
253.9 Chr09_32945967
269.3 Chr09_34813518

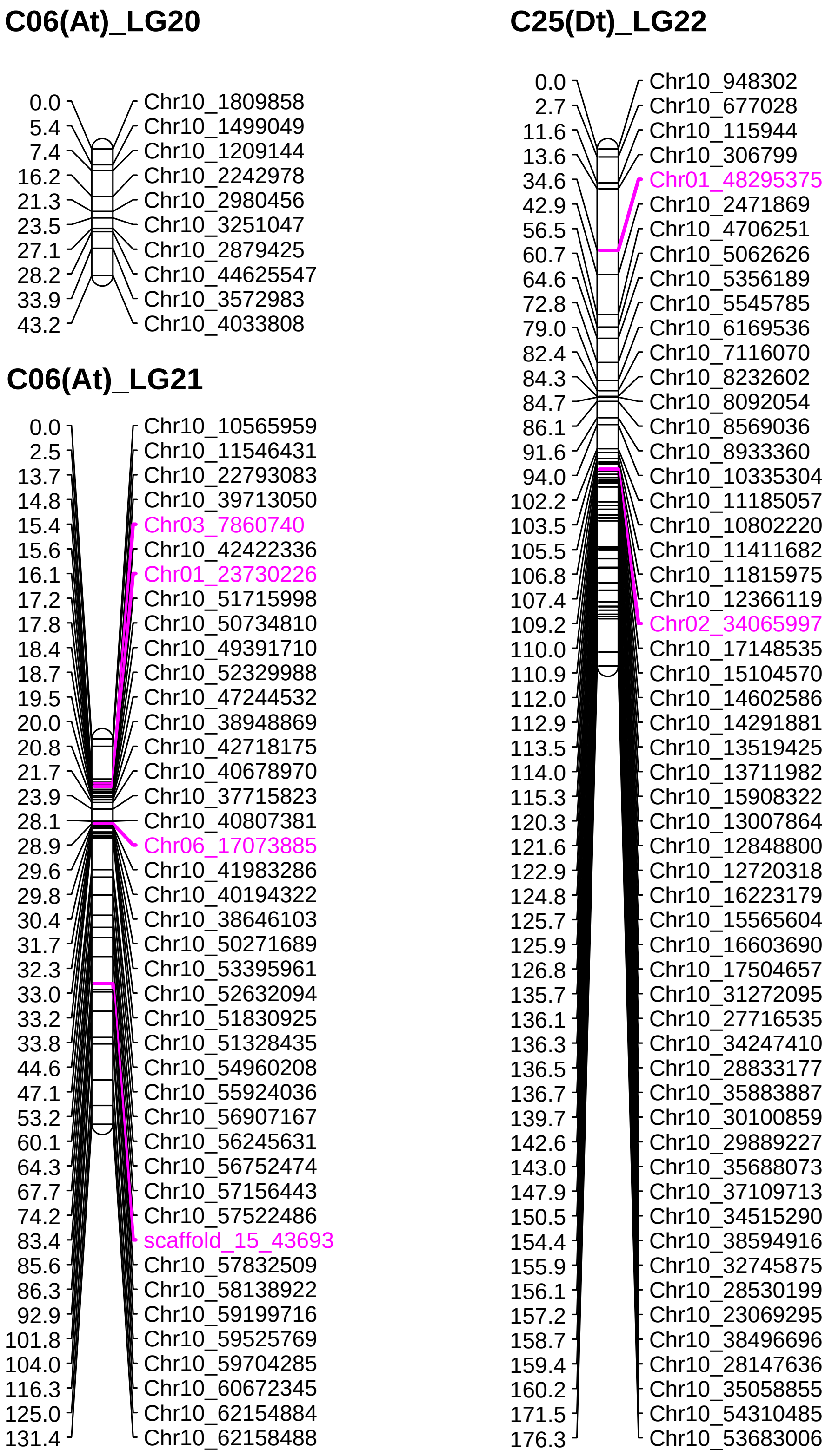
C06(At)_LG20
0.0 Chr10_1809858
5.4 Chr10_1499049
7.4 Chr10_1209144
16.2 Chr10_2242978
21.3 Chr10_2980456
23.5 Chr10_3251047
27.1 Chr10_2879425
28.2 Chr10_44625547
33.9 Chr10_3572983
43.2 Chr10_4033808
C06(At)_LG21
0.0 Chr10_10565959
2.5 Chr10_11546431
13.7 Chr10_22793083
14.8 Chr10_39713050
15.4 Chr03_7860740
15.6 Chr10_42422336
16.1 Chr01_23730226
17.2 Chr10_51715998
17.8 Chr10_50734810
18.4 Chr10_49391710
18.7 Chr10_52329988
19.5 Chr10_47244532
20.0 Chr10_38948869
20.8 Chr10_42718175
21.7 Chr10_40678970
23.9 Chr10_37715823
28.1 Chr10_40807381
28.9 Chr06_17073885
29.6 Chr10_41983286
29.8 Chr10_40194322
30.4 Chr10_38646103
31.7 Chr10_50271689
32.3 Chr10_53395961
33.0 Chr10_52632094
33.2 Chr10_51830925
33.8 Chr10_51328435
44.6 Chr10_54960208
47.1 Chr10_55924036
53.2 Chr10_56907167
60.1 Chr10_56245631
64.3 Chr10_56752474
67.7 Chr10_57156443
74.2 Chr10_57522486
83.4 scaffold_15_43693
85.6 Chr10_57832509
86.3 Chr10_58138922
92.9 Chr10_59199716
101.8 Chr10_59525769
104.0 Chr10_59704285
116.3 Chr10_60672345
125.0 Chr10_62154884
131.4 Chr10_62158488
C25(Dt)_LG22
0.0 Chr10_948302
2.7 Chr10_677028
11.6 Chr10_115944
13.6 Chr10_306799
34.6 Chr01_48295375
42.9 Chr10_2471869
56.5 Chr10_4706251
60.7 Chr10_5062626
64.6 Chr10_5356189
72.8 Chr10_5545785
79.0 Chr10_6169536
82.4 Chr10_7116070
84.3 Chr10_8232602
84.7 Chr10_8092054
86.1 Chr10_8569036
91.6 Chr10_8933360
94.0 Chr10_10335304
102.2 Chr10_11185057
103.5 Chr10_10802220
105.5 Chr10_11411682
106.8 Chr10_11815975
107.4 Chr10_12366119
109.2 Chr02_34065997
110.0 Chr10_17148535
110.9 Chr10_15104570
112.0 Chr10_14602586
112.9 Chr10_14291881
113.5 Chr10_13519425
114.0 Chr10_13711982
115.3 Chr10_15908322
120.3 Chr10_13007864
121.6 Chr10_12848800
122.9 Chr10_12720318
124.8 Chr10_16223179
125.7 Chr10_15565604
125.9 Chr10_16603690
126.8 Chr10_17504657
135.7 Chr10_31272095
136.1 Chr10_27716535
136.3 Chr10_34247410
136.5 Chr10_28833177
136.7 Chr10_35883887
139.7 Chr10_30100859
142.6 Chr10_29889227
143.0 Chr10_35688073
147.9 Chr10_37109713
150.5 Chr10_34515290
154.4 Chr10_38594916
155.9 Chr10_32745875
156.1 Chr10_28530199
157.2 Chr10_23069295
158.7 Chr10_38496696
159.4 Chr10_28147636
160.2 Chr10_35058855
171.5 Chr10_54310485
176.3 Chr10_53683006

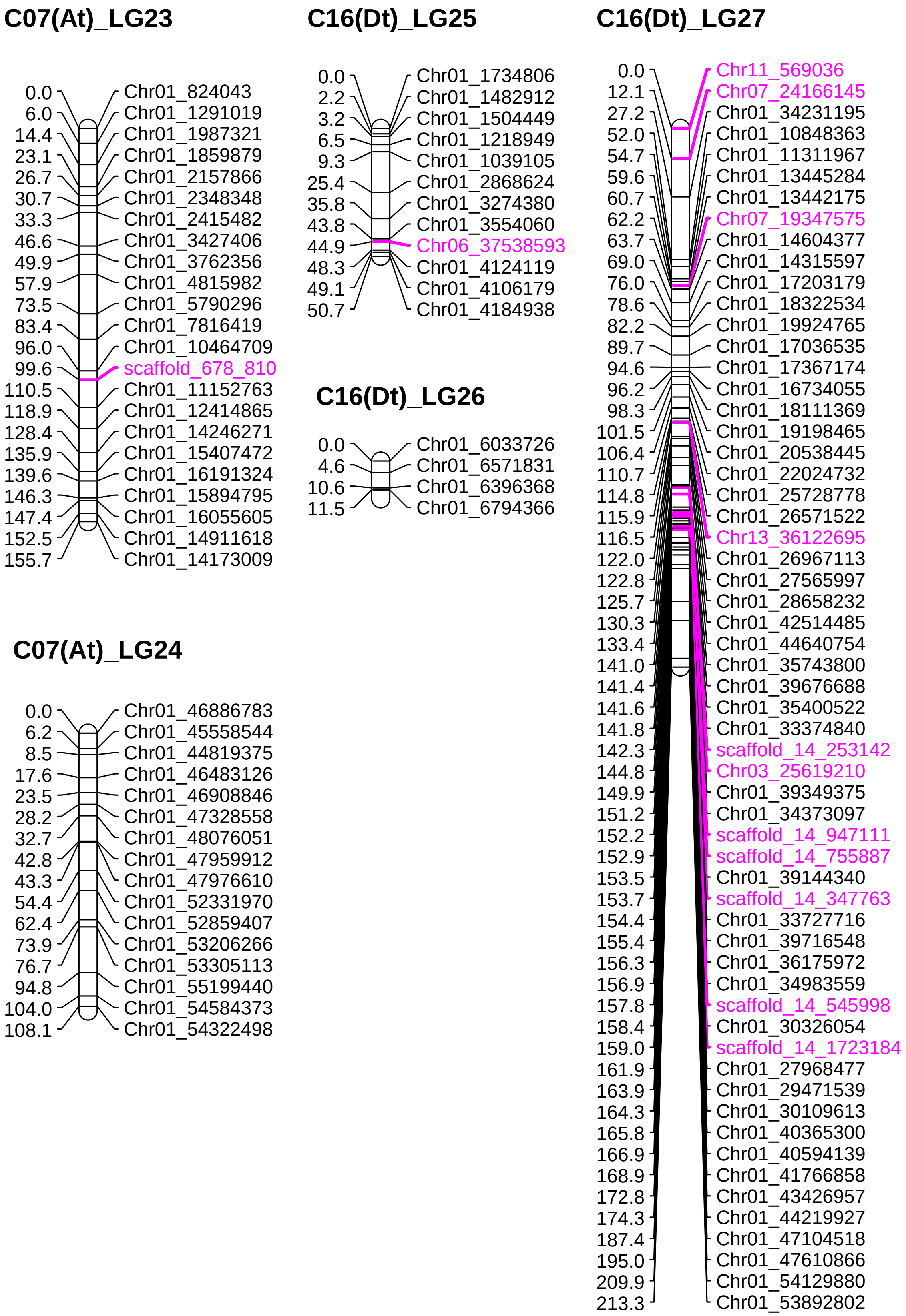
C07(At)_LG23
0.0 Chr01_824043
6.0 Chr01_1291019
14.4 Chr01_1987321
23.1 Chr01_1859879
26.7 Chr01_2157866
30.7 Chr01_2348348
33.3 Chr01_2415482
46.6 Chr01_3427406
49.9 Chr01_3762356
57.9 Chr01_4815982
73.5 Chr01_5790296
83.4 Chr01_7816419
96.0 Chr01_10464709
99.6 scaffold_678_810
110.5 Chr01_11152763
118.9 Chr01_12414865
128.4 Chr01_14246271
135.9 Chr01_15407472
139.6 Chr01_16191324
146.3 Chr01_15894795
147.4 Chr01_16055605
152.5 Chr01_14911618
155.7 Chr01_14173009
C07(At)_LG24
0.0 Chr01_46886783
6.2 Chr01_45558544
8.5 Chr01_44819375
17.6 Chr01_46483126
23.5 Chr01_46908846
28.2 Chr01_47328558
32.7 Chr01_48076051
42.8 Chr01_47959912
43.3 Chr01_47976610
54.4 Chr01_52331970
62.4 Chr01_52859407
73.9 Chr01_53206266
76.7 Chr01_53305113
94.8 Chr01_55199440
104.0 Chr01_54584373
108.1 Chr01_54322498
C16(Dt)_LG25
0.0 Chr01_1734806
2.2 Chr01_1482912
3.2 Chr01_1504449
6.5 Chr01_1218949
9.3 Chr01_1039105
25.4 Chr01_2868624
35.8 Chr01_3274380
43.8 Chr01_3554060
44.9 Chr06_37538593
48.3 Chr01_4124119
49.1 Chr01_4106179
50.7 Chr01_4184938
C16(Dt)_LG26
0.0 Chr01_6033726
4.6 Chr01_6571831
10.6 Chr01_6396368
11.5 Chr01_6794366
C16(Dt)_LG27
0.0 Chr11_569036
12.1 Chr07_24166145
27.2 Chr01_34231195
52.0 Chr01_10848363
54.7 Chr01_11311967
59.6 Chr01_13445284
60.7 Chr01_13442175
62.2 Chr07_19347575
63.7 Chr01_14604377
69.0 Chr01_14315597
76.0 Chr01_17203179
78.6 Chr01_18322534
82.2 Chr01_19924765
89.7 Chr01_17036535
94.6 Chr01_17367174
96.2 Chr01_16734055
98.3 Chr01_18111369
101.5 Chr01_19198465
106.4 Chr01_20538445
110.7 Chr01_22024732
114.8 Chr01_25728778
115.9 Chr01_26571522
116.5 Chr13_36122695
122.0 Chr01_26967113
122.8 Chr01_27565997
125.7 Chr01_28658232
130.3 Chr01_42514485
133.4 Chr01_44640754
141.0 Chr01_35743800
141.4 Chr01_39676688
141.6 Chr01_35400522
141.8 Chr01_33374840
142.3 scaffold_14_253142
144.8 Chr03_25619210
149.9 Chr01_39349375
151.2 Chr01_34373097
152.2 scaffold_14_947111
152.9 scaffold_14_755887
153.5 Chr01_39144340
153.7 scaffold_14_347763
154.4 Chr01_33727716
155.4 Chr01_39716548
156.3 Chr01_36175972
156.9 Chr01_34983559
157.8 scaffold_14_545998
158.4 Chr01_30326054
159.0 scaffold_14_1723184
161.9 Chr01_27968477
163.9 Chr01_29471539
164.3 Chr01_30109613
165.8 Chr01_40365300
166.9 Chr01_40594139
168.9 Chr01_41766858
172.8 Chr01_43426957
174.3 Chr01_44219927
187.4 Chr01_47104518
195.0 Chr01_47610866
209.9 Chr01_54129880
213.3 Chr01_53892802

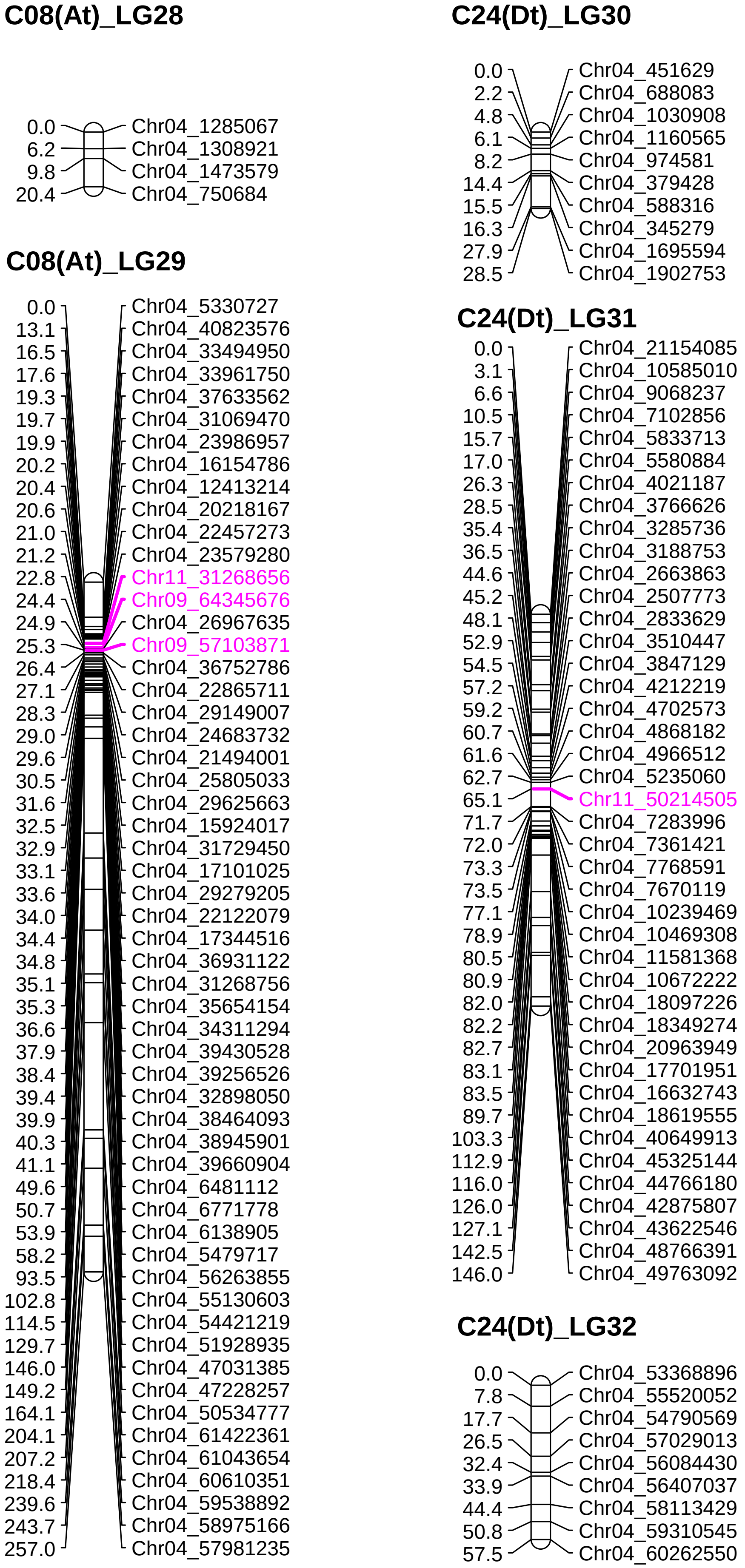
C08(At)_LG28
0.0 Chr04_1285067
6.2 Chr04_1308921
9.8 Chr04_1473579
20.4 Chr04_750684
C08(At)_LG29
0.0 Chr04_5330727
13.1 Chr04_40823576
16.5 Chr04_33494950
17.6 Chr04_33961750
19.3 Chr04_37633562
19.7 Chr04_31069470
19.9 Chr04_23986957
20.2 Chr04_16154786
20.4 Chr04_12413214
20.6 Chr04_20218167
21.0 Chr04_22457273
21.2 Chr04_23579280
22.8 Chr11_31268656
24.4 Chr09_64345676
24.9 Chr04_26967635
25.3 Chr09_57103871
26.4 Chr04_36752786
27.1 Chr04_22865711
28.3 Chr04_29149007
29.0 Chr04_24683732
29.6 Chr04_21494001
30.5 Chr04_25805033
31.6 Chr04_29625663
32.5 Chr04_15924017
32.9 Chr04_31729450
33.1 Chr04_17101025
33.6 Chr04_29279205
34.0 Chr04_22122079
34.4 Chr04_17344516
34.8 Chr04_36931122
35.1 Chr04_31268756
35.3 Chr04_35654154
36.6 Chr04_34311294
37.9 Chr04_39430528
38.4 Chr04_39256526
39.4 Chr04_32898050
39.9 Chr04_38464093
40.3 Chr04_38945901
41.1 Chr04_39660904
49.6 Chr04_6481112
50.7 Chr04_6771778
53.9 Chr04_6138905
58.2 Chr04_5479717
93.5 Chr04_56263855
102.8 Chr04_55130603
114.5 Chr04_54421219
129.7 Chr04_51928935
146.0 Chr04_47031385
149.2 Chr04_47228257
164.1 Chr04_50534777
204.1 Chr04_61422361
207.2 Chr04_61043654
218.4 Chr04_60610351
239.6 Chr04_59538892
243.7 Chr04_58975166
257.0 Chr04_57981235
C24(Dt)_LG30
0.0 Chr04_451629
2.2 Chr04_688083
4.8 Chr04_1030908
6.1 Chr04_1160565
8.2 Chr04_974581
14.4 Chr04_379428
15.5 Chr04_588316
16.3 Chr04_345279
27.9 Chr04_1695594
28.5 Chr04_1902753
C24(Dt)_LG31
0.0 Chr04_21154085
3.1 Chr04_10585010
6.6 Chr04_9068237
10.5 Chr04_7102856
15.7 Chr04_5833713
17.0 Chr04_5580884
26.3 Chr04_4021187
28.5 Chr04_3766626
35.4 Chr04_3285736
36.5 Chr04_3188753
44.6 Chr04_2663863
45.2 Chr04_2507773
48.1 Chr04_2833629
52.9 Chr04_3510447
54.5 Chr04_3847129
57.2 Chr04_4212219
59.2 Chr04_4702573
60.7 Chr04_4868182
61.6 Chr04_4966512
62.7 Chr04_5235060
65.1 Chr11_50214505
71.7 Chr04_7283996
72.0 Chr04_7361421
73.3 Chr04_7768591
73.5 Chr04_7670119
77.1 Chr04_10239469
78.9 Chr04_10469308
80.5 Chr04_11581368
80.9 Chr04_10672222
82.0 Chr04_18097226
82.2 Chr04_18349274
82.7 Chr04_20963949
83.1 Chr04_17701951
83.5 Chr04_16632743
89.7 Chr04_18619555
103.3 Chr04_40649913
112.9 Chr04_45325144
116.0 Chr04_44766180
126.0 Chr04_42875807
127.1 Chr04_43622546
142.5 Chr04_48766391
146.0 Chr04_49763092
C24(Dt)_LG32
0.0 Chr04_53368896
7.8 Chr04_55520052
17.7 Chr04_54790569
26.5 Chr04_57029013
32.4 Chr04_56084430
33.9 Chr04_56407037
44.4 Chr04_58113429
50.8 Chr04_59310545
57.5 Chr04_60262550

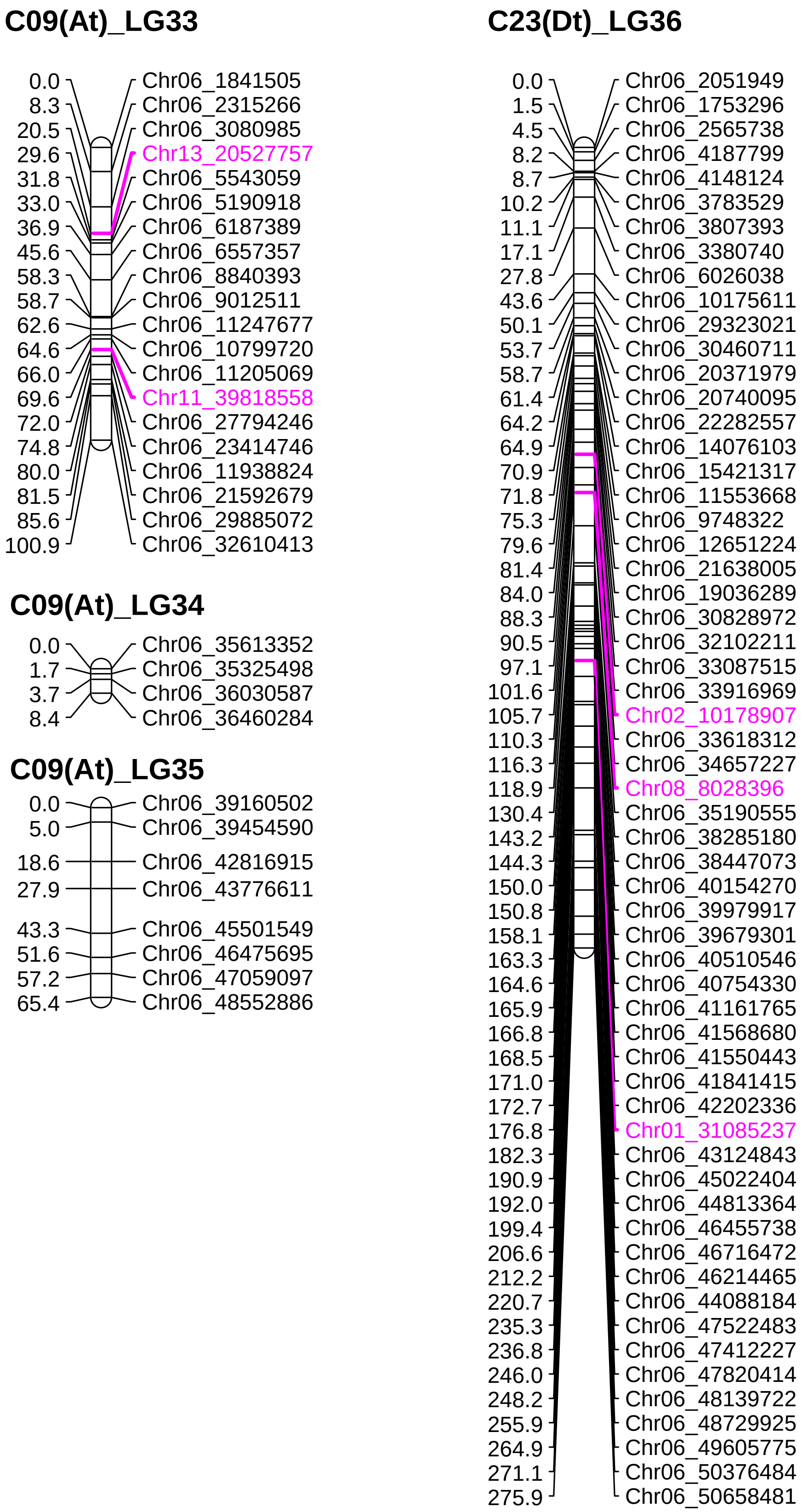
C09(At)_LG33
0.0 Chr06_1841505
8.3 Chr06_2315266
20.5 Chr06_3080985
29.6 Chr13_20527757
31.8 Chr06_5543059
33.0 Chr06_5190918
36.9 Chr06_6187389
45.6 Chr06_6557357
58.3 Chr06_8840393
58.7 Chr06_9012511
62.6 Chr06_11247677
64.6 Chr06_10799720
66.0 Chr06_11205069
69.6 Chr11_39818558
72.0 Chr06_27794246
74.8 Chr06_23414746
80.0 Chr06_11938824
81.5 Chr06_21592679
85.6 Chr06_29885072
100.9 Chr06_32610413
C09(At)_LG34
0.0 Chr06_35613352
1.7 Chr06_35325498
3.7 Chr06_36030587
8.4 Chr06_36460284
C09(At)_LG35
0.0 Chr06_39160502
5.0 Chr06_39454590
18.6 Chr06_42816915
27.9 Chr06_43776611
43.3 Chr06_45501549
51.6 Chr06_46475695
57.2 Chr06_47059097
65.4 Chr06_48552886
C23(Dt)_LG36
0.0 Chr06_2051949
1.5 Chr06_1753296
4.5 Chr06_2565738
8.2 Chr06_4187799
8.7 Chr06_4148124
10.2 Chr06_3783529
11.1 Chr06_3807393
17.1 Chr06_3380740
27.8 Chr06_6026038
43.6 Chr06_10175611
50.1 Chr06_29323021
53.7 Chr06_30460711
58.7 Chr06_20371979
61.4 Chr06_20740095
64.2 Chr06_22282557
64.9 Chr06_14076103
70.9 Chr06_15421317
71.8 Chr06_11553668
75.3 Chr06_9748322
79.6 Chr06_12651224
81.4 Chr06_21638005
84.0 Chr06_19036289
88.3 Chr06_30828972
90.5 Chr06_32102211
97.1 Chr06_33087515
101.6 Chr06_33916969
105.7 Chr02_10178907
110.3 Chr06_33618312
116.3 Chr06_34657227
118.9 Chr08_8028396
130.4 Chr06_35190555
143.2 Chr06_38285180
144.3 Chr06_38447073
150.0 Chr06_40154270
150.8 Chr06_39979917
158.1 Chr06_39679301
163.3 Chr06_40510546
164.6 Chr06_40754330
165.9 Chr06_41161765
166.8 Chr06_41568680
168.5 Chr06_41550443
171.0 Chr06_41841415
172.7 Chr06_42202336
176.8 Chr01_31085237
182.3 Chr06_43124843
190.9 Chr06_45022404
192.0 Chr06_44813364
199.4 Chr06_46455738
206.6 Chr06_46716472
212.2 Chr06_46214465
220.7 Chr06_44088184
235.3 Chr06_47522483
236.8 Chr06_47412227
246.0 Chr06_47820414
248.2 Chr06_48139722
255.9 Chr06_48729925
264.9 Chr06_49605775
271.1 Chr06_50376484
275.9 Chr06_50658481

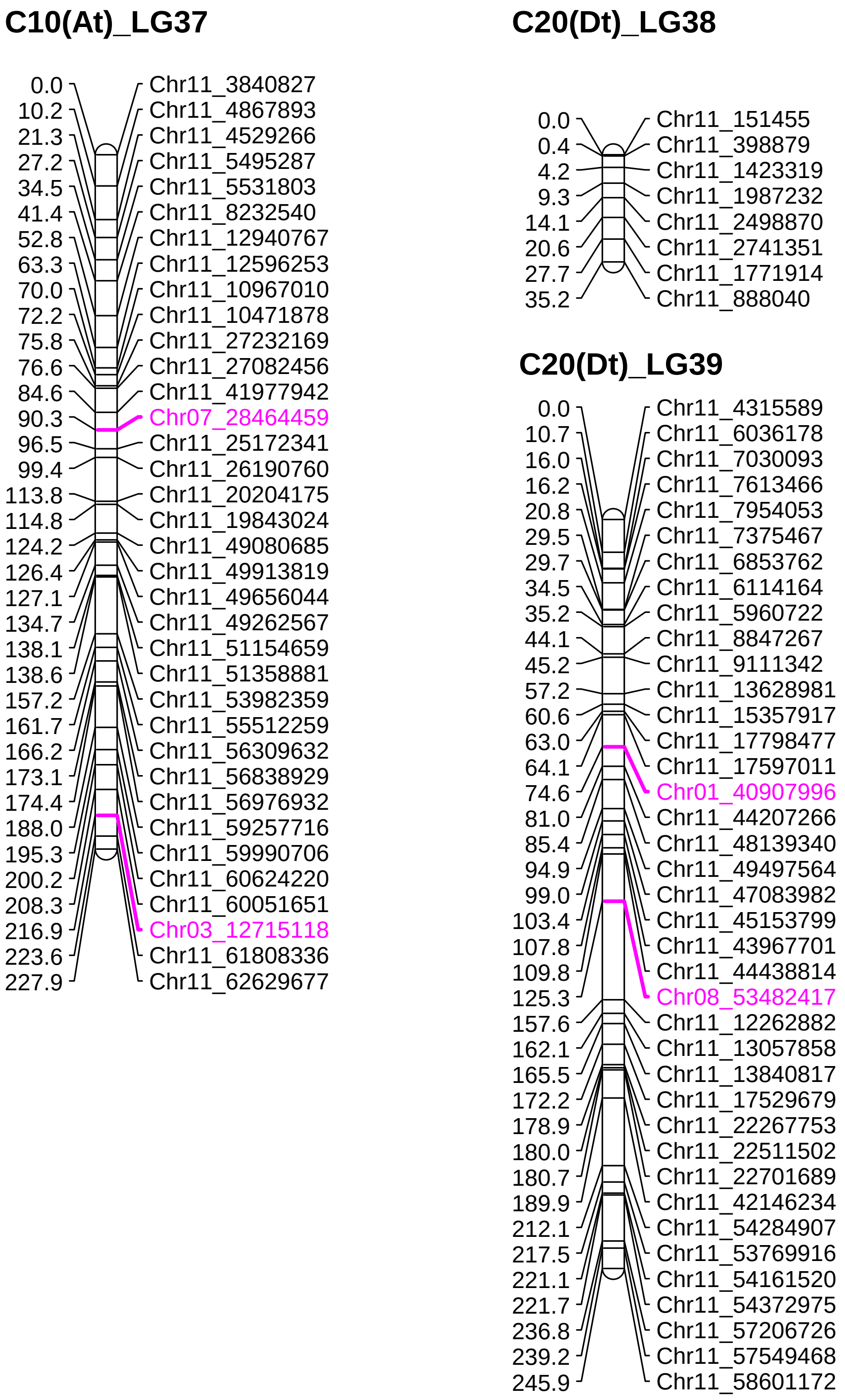
C10(At)_LG37
0.0 Chr11_3840827
10.2 Chr11_4867893
21.3 Chr11_4529266
27.2 Chr11_5495287
34.5 Chr11_5531803
41.4 Chr11_8232540
52.8 Chr11_12940767
63.3 Chr11_12596253
70.0 Chr11_10967010
72.2 Chr11_10471878
75.8 Chr11_27232169
76.6 Chr11_27082456
84.6 Chr11_41977942
90.3 Chr07_28464459
96.5 Chr11_25172341
99.4 Chr11_26190760
113.8 Chr11_20204175
114.8 Chr11_19843024
124.2 Chr11_49080685
126.4 Chr11_49913819
127.1 Chr11_49656044
134.7 Chr11_49262567
138.1 Chr11_51154659
138.6 Chr11_51358881
157.2 Chr11_53982359
161.7 Chr11_55512259
166.2 Chr11_56309632
173.1 Chr11_56838929
174.4 Chr11_56976932
188.0 Chr11_59257716
195.3 Chr11_59990706
200.2 Chr11_60624220
208.3 Chr11_60051651
216.9 Chr03_12715118
223.6 Chr11_61808336
227.9 Chr11_62629677
C20(Dt)_LG38
0.0 Chr11_151455
0.4 Chr11_398879
4.2 Chr11_1423319
9.3 Chr11_1987232
14.1 Chr11_2498870
20.6 Chr11_2741351
27.7 Chr11_1771914
35.2 Chr11_888040
C20(Dt)_LG39
0.0 Chr11_4315589
10.7 Chr11_6036178
16.0 Chr11_7030093
16.2 Chr11_7613466
20.8 Chr11_7954053
29.5 Chr11_7375467
29.7 Chr11_6853762
34.5 Chr11_6114164
35.2 Chr11_5960722
44.1 Chr11_8847267
45.2 Chr11_9111342
57.2 Chr11_13628981
60.6 Chr11_15357917
63.0 Chr11_17798477
64.1 Chr11_17597011
74.6 Chr01_40907996
81.0 Chr11_44207266
85.4 Chr11_48139340
94.9 Chr11_49497564
99.0 Chr11_47083982
103.4 Chr11_45153799
107.8 Chr11_43967701
109.8 Chr11_44438814
125.3 Chr08_53482417
157.6 Chr11_12262882
162.1 Chr11_13057858
165.5 Chr11_13840817
172.2 Chr11_17529679
178.9 Chr11_22267753
180.0 Chr11_22511502
180.7 Chr11_22701689
189.9 Chr11_42146234
212.1 Chr11_54284907
217.5 Chr11_53769916
221.1 Chr11_54161520
221.7 Chr11_54372975
236.8 Chr11_57206726
239.2 Chr11_57549468
245.9 Chr11_58601172

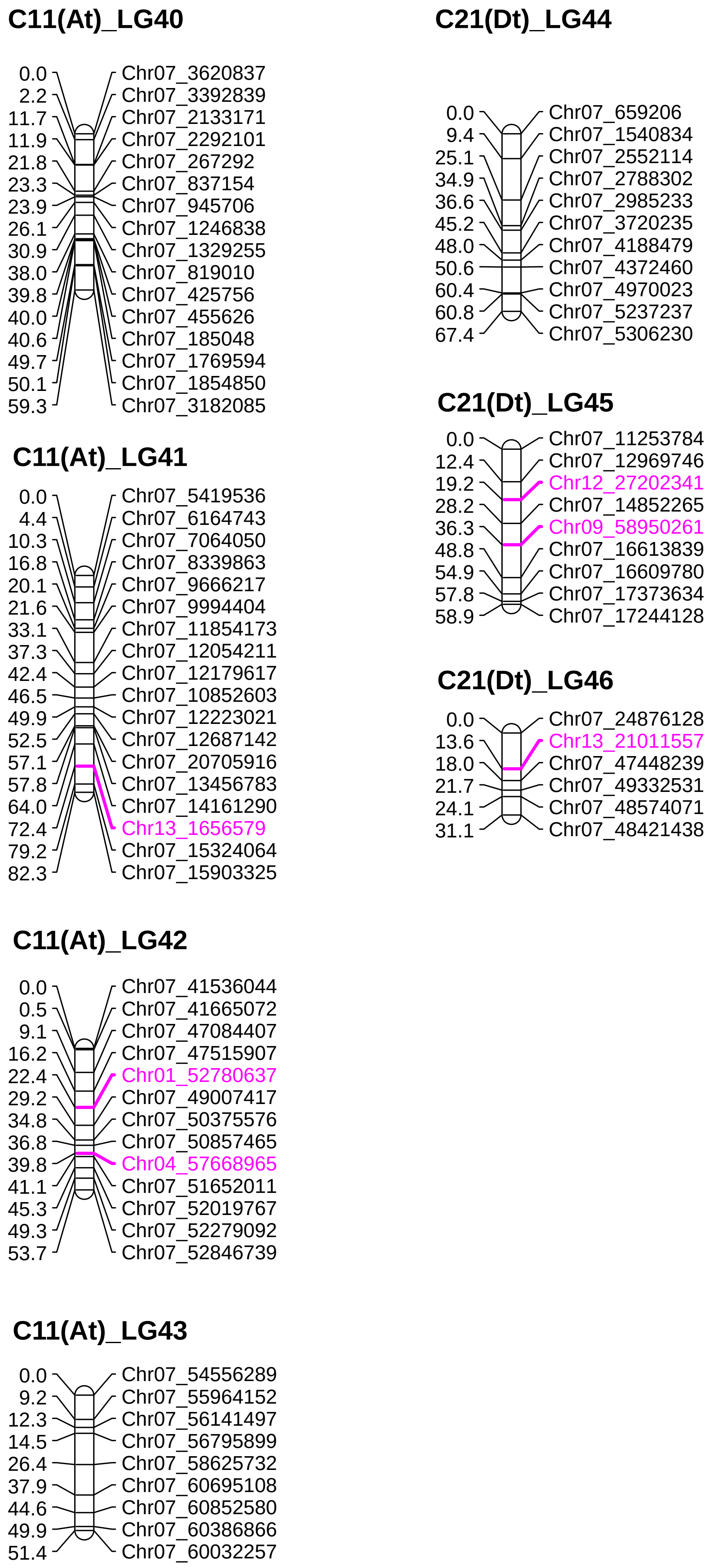
C11(At)_LG40
0.0 Chr07_3620837
2.2 Chr07_3392839
11.7 Chr07_2133171
11.9 Chr07_2292101
21.8 Chr07_267292
23.3 Chr07_837154
23.9 Chr07_945706
26.1 Chr07_1246838
30.9 Chr07_1329255
38.0 Chr07_819010
39.8 Chr07_425756
40.0 Chr07_455626
40.6 Chr07_185048
49.7 Chr07_1769594
50.1 Chr07_1854850
59.3 Chr07_3182085
C11(At)_LG41
0.0 Chr07_5419536
4.4 Chr07_6164743
10.3 Chr07_7064050
16.8 Chr07_8339863
20.1 Chr07_9666217
21.6 Chr07_9994404
33.1 Chr07_11854173
37.3 Chr07_12054211
42.4 Chr07_12179617
46.5 Chr07_10852603
49.9 Chr07_12223021
52.5 Chr07_12687142
57.1 Chr07_20705916
57.8 Chr07_13456783
64.0 Chr07_14161290
72.4 Chr13_1656579
79.2 Chr07_15324064
82.3 Chr07_15903325
C11(At)_LG42
0.0 Chr07_41536044
0.5 Chr07_41665072
9.1 Chr07_47084407
16.2 Chr07_47515907
22.4 Chr01_52780637
29.2 Chr07_49007417
34.8 Chr07_50375576
36.8 Chr07_50857465
39.8 Chr04_57668965
41.1 Chr07_51652011
45.3 Chr07_52019767
49.3 Chr07_52279092
53.7 Chr07_52846739
C11(At)_LG43
0.0 Chr07_54556289
9.2 Chr07_55964152
12.3 Chr07_56141497
14.5 Chr07_56795899
26.4 Chr07_58625732
37.9 Chr07_60695108
44.6 Chr07_60852580
49.9 Chr07_60386866
51.4 Chr07_60032257
C21(Dt)_LG44
0.0 Chr07_659206
9.4 Chr07_1540834
25.1 Chr07_2552114
34.9 Chr07_2788302
36.6 Chr07_2985233
45.2 Chr07_3720235
48.0 Chr07_4188479
50.6 Chr07_4372460
60.4 Chr07_4970023
60.8 Chr07_5237237
67.4 Chr07_5306230
C21(Dt)_LG45
0.0 Chr07_11253784
12.4 Chr07_12969746
19.2 Chr12_27202341
28.2 Chr07_14852265
36.3 Chr09_58950261
48.8 Chr07_16613839
54.9 Chr07_16609780
57.8 Chr07_17373634
58.9 Chr07_17244128
C21(Dt)_LG46
0.0 Chr07_24876128
13.6 Chr13_21011557
18.0 Chr07_47448239
21.7 Chr07_49332531
24.1 Chr07_48574071
31.1 Chr07_48421438

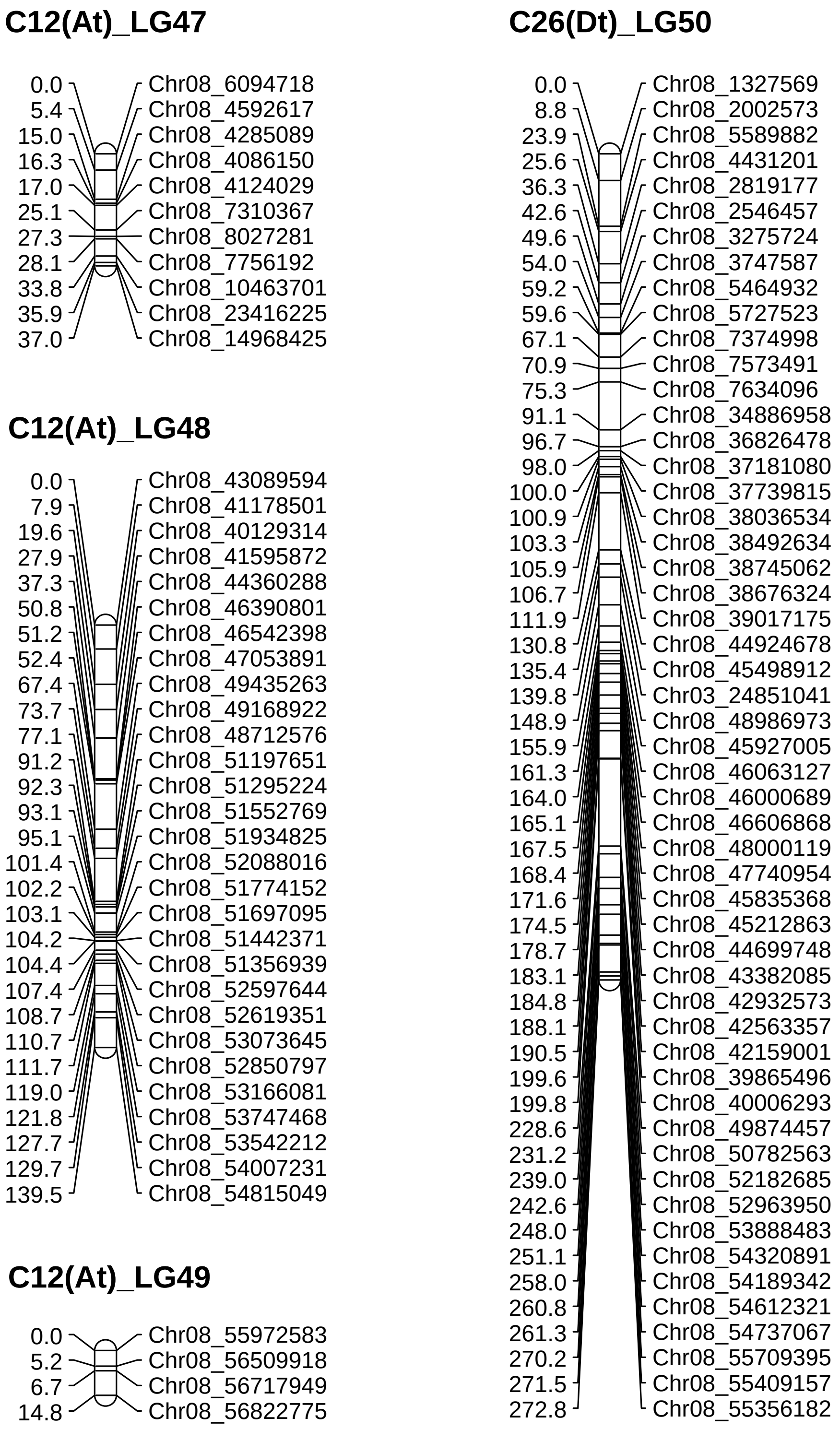

C12(At)_LG47
0.0 Chr08_6094718
5.4 Chr08_4592617
15.0 Chr08_4285089
16.3 Chr08_4086150
17.0 Chr08_4124029
25.1 Chr08_7310367
27.3 Chr08_8027281
28.1 Chr08_7756192
33.8 Chr08_10463701
35.9 Chr08_23416225
37.0 Chr08_14968425
C12(At)_LG48
0.0 Chr08_43089594
7.9 Chr08_41178501
19.6 Chr08_40129314
27.9 Chr08_41595872
37.3 Chr08_44360288
50.8 Chr08_46390801
51.2 Chr08_46542398
52.4 Chr08_47053891
67.4 Chr08_49435263
73.7 Chr08_49168922
77.1 Chr08_48712576
91.2 Chr08_51197651
92.3 Chr08_51295224
93.1 Chr08_51552769
95.1 Chr08_51934825
101.4 Chr08_52088016
102.2 Chr08_51774152
103.1 Chr08_51697095
104.2 Chr08_51442371
104.4 Chr08_51356939
107.4 Chr08_52597644
108.7 Chr08_52619351
110.7 Chr08_53073645
111.7 Chr08_52850797
119.0 Chr08_53166081
121.8 Chr08_53747468
127.7 Chr08_53542212
129.7 Chr08_54007231
139.5 Chr08_54815049
C12(At)_LG49
0.0 Chr08_55972583
5.2 Chr08_56509918
6.7 Chr08_56717949
14.8 Chr08_56822775
C26(Dt)_LG50
0.0 Chr08_1327569
8.8 Chr08_2002573
23.9 Chr08_5589882
25.6 Chr08_4431201
36.3 Chr08_2819177
42.6 Chr08_2546457
49.6 Chr08_3275724
54.0 Chr08_3747587
59.2 Chr08_5464932
59.6 Chr08_5727523
67.1 Chr08_7374998
70.9 Chr08_7573491
75.3 Chr08_7634096
91.1 Chr08_34886958
96.7 Chr08_36826478
98.0 Chr08_37181080
100.0 Chr08_37739815
100.9 Chr08_38036534
103.3 Chr08_38492634
105.9 Chr08_38745062
106.7 Chr08_38676324
111.9 Chr08_39017175
130.8 Chr08_44924678
135.4 Chr08_45498912
139.8 Chr03_24851041
148.9 Chr08_48986973
155.9 Chr08_45927005
161.3 Chr08_46063127
164.0 Chr08_46000689
165.1 Chr08_46606868
167.5 Chr08_48000119
168.4 Chr08_47740954
171.6 Chr08_45835368
174.5 Chr08_45212863
178.7 Chr08_44699748
183.1 Chr08_43382085
184.8 Chr08_42932573
188.1 Chr08_42563357
190.5 Chr08_42159001
199.6 Chr08_39865496
199.8 Chr08_40006293
228.6 Chr08_49874457
231.2 Chr08_50782563
239.0 Chr08_52182685
242.6 Chr08_52963950
248.0 Chr08_53888483
251.1 Chr08_54320891
258.0 Chr08_54189342
260.8 Chr08_54612321
261.3 Chr08_54737067
270.2 Chr08_55709395
271.5 Chr08_55409157
272.8 Chr08_55356182

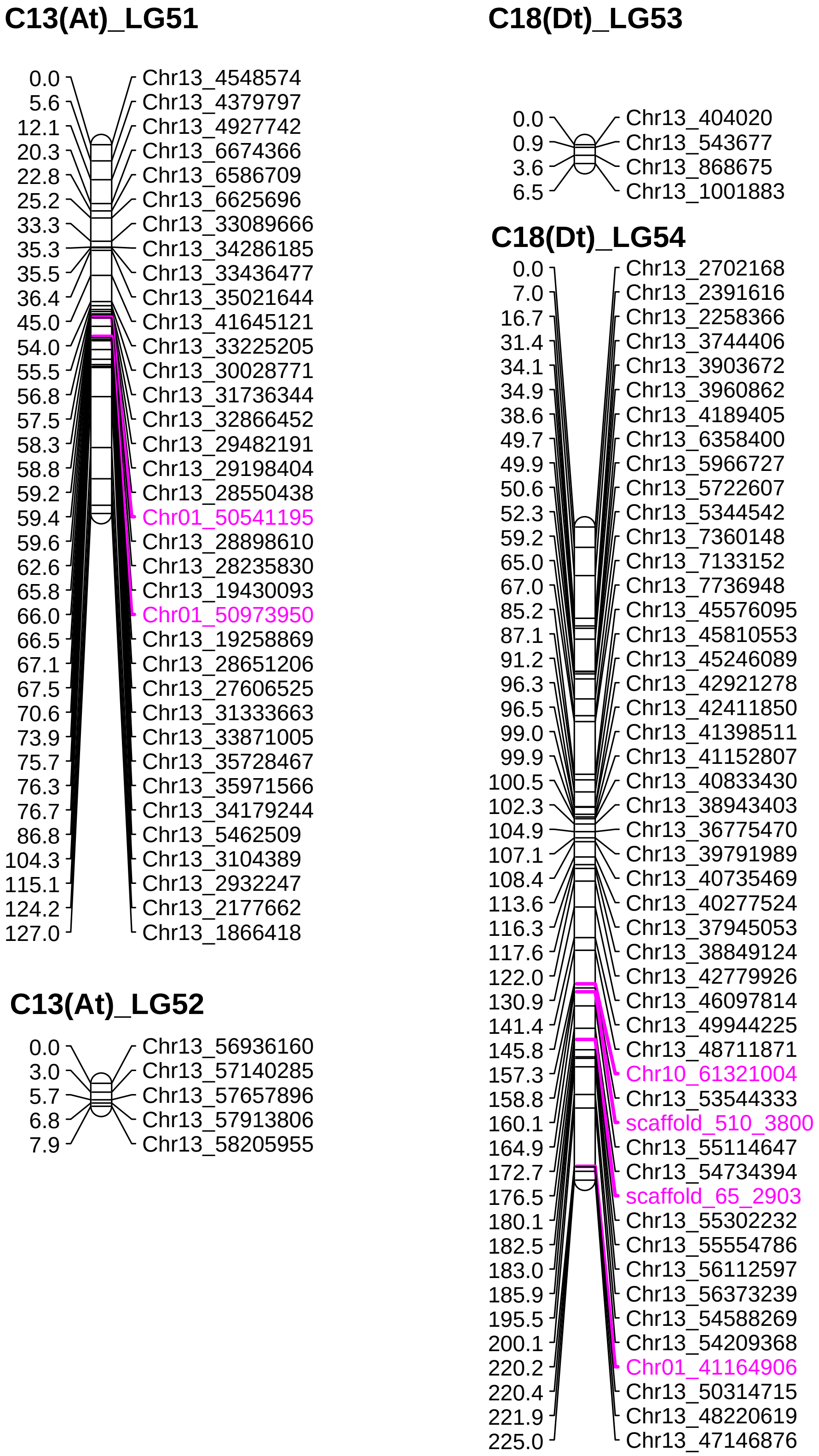
C13(At)_LG51
0.0 Chr13_4548574
5.6 Chr13_4379797
12.1 Chr13_4927742
20.3 Chr13_6674366
22.8 Chr13_6586709
25.2 Chr13_6625696
33.3 Chr13_33089666
35.3 Chr13_34286185
35.5 Chr13_33436477
36.4 Chr13_35021644
45.0 Chr13_41645121
54.0 Chr13_33225205
55.5 Chr13_30028771
56.8 Chr13_31736344
57.5 Chr13_32866452
58.3 Chr13_29482191
58.8 Chr13_29198404
59.2 Chr13_28550438
59.4 Chr01_50541195
59.6 Chr13_28898610
62.6 Chr13_28235830
65.8 Chr13_19430093
66.0 Chr01_50973950
66.5 Chr13_19258869
67.1 Chr13_28651206
67.5 Chr13_27606525
70.6 Chr13_31333663
73.9 Chr13_33871005
75.7 Chr13_35728467
76.3 Chr13_35971566
76.7 Chr13_34179244
86.8 Chr13_5462509
104.3 Chr13_3104389
115.1 Chr13_2932247
124.2 Chr13_2177662
127.0 Chr13_1866418
C13(At)_LG52
0.0 Chr13_56936160
3.0 Chr13_57140285
5.7 Chr13_57657896
6.8 Chr13_57913806
7.9 Chr13_58205955
C18(Dt)_LG53
0.0 Chr13_404020
0.9 Chr13_543677
3.6 Chr13_868675
6.5 Chr13_1001883
C18(Dt)_LG54
0.0 Chr13_2702168
7.0 Chr13_2391616
16.7 Chr13_2258366
31.4 Chr13_3744406
34.1 Chr13_3903672
34.9 Chr13_3960862
38.6 Chr13_4189405
49.7 Chr13_6358400
49.9 Chr13_5966727
50.6 Chr13_5722607
52.3 Chr13_5344542
59.2 Chr13_7360148
65.0 Chr13_7133152
67.0 Chr13_7736948
85.2 Chr13_45576095
87.1 Chr13_45810553
91.2 Chr13_45246089
96.3 Chr13_42921278
96.5 Chr13_42411850
99.0 Chr13_41398511
99.9 Chr13_41152807
100.5 Chr13_40833430
102.3 Chr13_38943403
104.9 Chr13_36775470
107.1 Chr13_39791989
108.4 Chr13_40735469
113.6 Chr13_40277524
116.3 Chr13_37945053
117.6 Chr13_38849124
122.0 Chr13_42779926
130.9 Chr13_46097814
141.4 Chr13_49944225
145.8 Chr13_48711871
157.3 Chr10_61321004
158.8 Chr13_53544333
160.1 scaffold_510_3800
164.9 Chr13_55114647
172.7 Chr13_54734394
176.5 scaffold_65_2903
180.1 Chr13_55302232
182.5 Chr13_55554786
183.0 Chr13_56112597
185.9 Chr13_56373239
195.5 Chr13_54588269
200.1 Chr13_54209368
220.2 Chr01_41164906
220.4 Chr13_50314715
221.9 Chr13_48220619
225.0 Chr13_47146876
